# An individual participant data meta-analysis investigating the mediating role of eating behavior traits in Acceptance and Commitment Therapy-based weight management interventions

**DOI:** 10.1093/abm/kaaf039

**Published:** 2025-06-02

**Authors:** Laura Kudlek, Julia Mueller, Patricia Eustachio Colombo, Stephen J Sharp, Clare E Boothby, Simon J Griffin, Meghan Butryn, Christina Chwyl, Evan Forman, Charlotte Hagerman, Misty Hawkins, Adrienne Juarascio, Bärbel Knäuper, Marjukka Kolehmainen, Michael E Levin, Jason Lillis, Edurne Maiz, Stephanie Manasse, Lara Palmeira, Kirsi H Pietiläinen, Nancy E Sherwood, Amy Ahern

**Affiliations:** MRC Epidemiology Unit, University of Cambridge, Cambridge, CB2 0QQ, United Kingdom; MRC Epidemiology Unit, University of Cambridge, Cambridge, CB2 0QQ, United Kingdom; MRC Epidemiology Unit, University of Cambridge, Cambridge, CB2 0QQ, United Kingdom; MRC Epidemiology Unit, University of Cambridge, Cambridge, CB2 0QQ, United Kingdom; MRC Epidemiology Unit, University of Cambridge, Cambridge, CB2 0QQ, United Kingdom; MRC Epidemiology Unit, University of Cambridge, Cambridge, CB2 0QQ, United Kingdom; Department of Psychology, Drexel University, Philadelphia, PA 19104, United States; Department of Psychology, Drexel University, Philadelphia, PA 19104, United States; Department of Psychology, Drexel University, Philadelphia, PA 19104, United States; Department of Psychology, Drexel University, Philadelphia, PA 19104, United States; Department of Health and Wellness Design, School of Public Health, Indiana University, Bloomington, IN 47405, United States; Department of Psychology, Drexel University, Philadelphia, PA 19104, United States; Department of Psychology, McGill University, Montreal, Quebec, H3A 1G1, Canada; Institute of Public Health and Clinical Nutrition, University of Eastern Finland, Kuopio, FI-70211, Finland; Department of Psychology, Utah State University, Logan, UT 84322, United States; The Department of Psychiatry and Human Behavior, Warren Alpert Medical School of Brown University, Providence, RI 02912, United States; The Weight Control and Diabetes Research Center, The Miriam Hospital, Providence, RI 02903, United States; Department of Clinical and Health Psychology and Research Methodology, University of the Basque Country UPV/EHU, Donostia-San Sebastian, Basque Country, 20018, Spain; Department of Psychology, Drexel University, Philadelphia, PA 19104, United States; RISE-Health, Department of Psychology and Education, Universidade Portucalense Infante D. Henrique, Rua Dr. António Bernardino de Almeida, Porto, 4200-319, Portugal; Obesity Research Unit, Research Program for Clinical and Molecular Metabolism, Faculty of Medicine, University of Helsinki, Helsinki, FI-00014, Finland; Healthy Weight Hub, Abdominal Center, Helsinki University Hospital, Helsinki, 00290, Finland; Division of Epidemiology and Community Health, School of Public Health, University of Minnesota, Minneapolis, MN 55454, United States; MRC Epidemiology Unit, University of Cambridge, Cambridge, CB2 0QQ, United Kingdom

**Keywords:** eating behavior traits, weight management, Acceptance and Commitment Therapy, mechanisms of action

## Abstract

**Background:**

Identifying mechanisms of action can aid the refinement of weight management interventions. Acceptance and Commitment Therapy (ACT)-based interventions may support long-term weight management by improving self-regulation of eating behavior traits (EBTs). However, it remains unclear if changing EBTs like emotional eating, external eating, internal disinhibition, and restraint during ACT causes improved weight management.

**Methods:**

For this 1-stage Individual Participant Data (IPD) meta-analysis, we requested IPD from 9 trials identified through a systematic search of ACT-based interventions for adults with a body mass index >25 kg/m^2^ across 8 databases until June 20, 2022. We obtained, checked, and harmonized data from 8 of those trials (*N* = 1391) and conducted separate structural equation models with complex survey analysis to estimate short- and long-term mediating effects of changes in each EBT on percent weight change.

**Results:**

In the short-term (ie, follow-up closest to intervention end), we found indirect effects of the intervention on percent weight change through changes in emotional eating, external eating, internal disinhibition, and restraint. Each 1-unit change in these EBTs led to a 0.02% (95% CI, 0.05–0.001), 0.03% (95% CI, 0.06–0.001), 0.05% (95% CI, 0.11–0.02), and 0.09% (95% CI, 0.14–0.04) decrease in weight, respectively. In the long term (ie, 12 months after intervention end), we found both indirect and total effects for emotional eating, internal disinhibition, and restraint, with EBT changes explaining 23.78%, 23.12%, and 25.64% of total effects.

**Conclusion:**

Findings suggest small partial mediating effects of ACT on weight through EBTs. Targeting EBTs may support improved weight management outcomes, particularly in the long term.

## Teaser

Acceptance and Commitment Therapy-based weight management programs supported weight loss, in part, through changes in eating behavior traits like emotional eating.

## Introduction

Overweight and obesity are highly prevalent and significant risk factors for numerous noncommunicable diseases, such as coronary heart disease, diabetes, hypertension, stroke, and several types of cancer.^1,2^ Behavioral weight management interventions (BWMIs) can reduce bodyweight and improve health.^3–5^ However, weight regain remains a concern^6–8^ and not all patients benefit equally from treatment.^9^ Identifying mechanisms that contribute to greater weight reductions and can be targeted in BWMIs could inform the development of improved treatments.

Eating behavior traits (EBTs), which are defined as individuals’ psycho-behavioral patterns around food and food-related cues,^10,11^ are considered significant contributors to obesity^12–19^ and weight management.^20–22^ Examples include restraint (deliberately limiting food intake to control body weight), internal disinhibition (loss of control eating in response to internal events like thoughts and feelings), external eating or external disinhibition (overeating in response to food cues in the environment), and emotional eating (overeating in response to negative emotions).^23–25^ EBTs typically change in the desired direction during BWMIs, including increases in restraint and decreases in emotional and uncontrolled eating.^26–30^ In turn, reductions in EBTs like emotional eating were associated with weight loss in several BWMIs.^27,31–34^ Few studies investigated whether changes in EBTs are causal of improved weight loss in formal mediation analyses, but initial evidence suggests BWMIs can lead to weight loss through reductions in emotional eating and disinhibition and increases in restraint, highlighting the importance of targeting these EBTs in treatment.^35–39^

Mediating effects, indicating the effect of an intervention on weight outcomes through changes in a mechanism of action, may vary across different types of treatment. Studies that investigated mediating effects of EBTs so far were mostly performed in standard BWMIs,^36–39^ which typically involve education and behavior change techniques aimed at modifying dietary and physical activity behaviors, such as self-monitoring, goal setting, planning, problem solving, or cognitive restructuring.^40^ While these standard BWMIs are considered to work well to address EBTs like restraint,^30^ it has been suggested that interventions based on Acceptance and Commitment Therapy (ACT) may better address dysregulated EBTs such as emotional eating and disinhibition.^41,42^

ACT aims to increase psychological flexibility and reduce experiential avoidance by encouraging present moment awareness and acceptance of unpleasant internal states, including thoughts, feelings, and sensations.^43^ ACT strategies can thus help individuals to recognize triggers of overeating and accept uncomfortable emotions and sensations such as cravings. This could reduce behavioral tendencies where individuals rely on food to manage urges and regulate emotions, including emotional eating, disinhibition, and external eating.^44^ Recent systematic reviews and meta-analyses concluded that ACT-based BWMIs are effective at reducing emotional eating^45^ and have better long-term weight management outcomes than standard BWMIs.^46^ However, to our knowledge, no studies have yet examined whether ACT-based interventions influence weight loss outcomes through changes in EBTs (ie, mediation).

This lack of evidence may stem from typically small sample sizes in ACT trials, which do not provide sufficient power to explore mediating effects in appropriate analyses. Statistical power can be improved in an Individual Participant Data (IPD) meta-analysis, which collects, harmonizes, and re-analyses raw data from any relevant trials, thereby increasing the sample size for analysis.^47^ Unlike a traditional aggregate meta-analysis, this method does not rely on summarizing existing findings but generates a secondary database to perform original research and answer novel research questions.

In this study, we aimed to explore whether and to what extent changes in EBTs mediate the effect of ACT-based interventions on weight loss outcomes in an IPD meta-analysis.

## Methods

We adhered to the guidelines outlined in the Preferred Reporting Items for Systematic Reviews and Meta-Analyses for IPD extension (PRISMA-IPD)^48^ and the “A Guideline for Reporting Mediation Analyses” (AGReMA),^49^ as detailed in Supplementary Sections 1.0 and 2.0). The study was preregistered on PROSPERO (CRD42022359691), with a study protocol published in *BMJ Open*.^50^ We also submitted an analysis plan to the Open Science Forum (OSF) prior to commencing analyses (doi: 10.17605/OSF.IO/3X6YM).

### Study identification and selection

We included both published and unpublished randomized controlled trials (RCTs) on adults ≥18 years with a body mass index (BMI) ≥25 kg/m^2^ who participated in an ACT-based intervention aimed at weight loss or weight loss maintenance. Trials needed to have data on EBTs at baseline and end of intervention and data on bodyweight at baseline and at any follow-up after intervention end. Eligible EBTs were emotional eating, external eating, disinhibition (general disinhibition, internal disinhibition, external disinhibition), restraint (general restraint, flexible restraint, rigid restraint), and uncontrolled eating, and were investigated if more than 5 included trials contributed data to them. Trials with a waitlist comparison group were excluded as they could have been receiving the ACT-based intervention at follow-up. Further details on participants, interventions, comparisons, outcomes, and study design (PICOs) are described in Supplementary Table 3-1.

We based our search strategy on a review by Lawlor et al.,^46^ which synthesized outcomes of third-wave cognitive behavioral therapies for weight management. We adapted their search by dropping concepts related to treatments other than ACT and ran it across 8 databases (MEDLINE, CINAHL, Embase, PsycINFO, AMED, ASSIA, Web of Science, and CENTRAL) up to June 20, 2022. In addition, we manually searched reference lists of key publications (see Supplementary Section 4.0 for the full search strategy).

Studies previously included in Lawlor et al. as well as newly identified records were screened in duplicate by P.E.C. and L.K. using Covidence.^51^ We resolved disagreements by discussion and consulted a third reviewer, A.A., where necessary. If the eligibility of a trial was unclear or the results not published yet, we contacted the trial authors to determine inclusion.

### Data collection and management

#### Requesting and collecting IPD

We emailed authors to invite them to collaborate and share their IPD. We sent 2 reminders approximately 3 weeks apart. If we did not receive a response or lost contact, we excluded the study. We established data transfer agreements (DTAs) with the institutions of collaborating authors and provided a detailed data dictionary of requested variables. These included participant ID, age, sex/gender, height, weight, EBTs, experiential avoidance, trial arm, number of sessions attended, and any exclusions and reasons for exclusion. While we acknowledge the ongoing discussions around the use of bodyweight as an outcome variable,^52–54^ we anticipated it to be the most commonly measured variable across eligible trials and thus the one that would allow for the inclusion of the greatest number of studies. Variables were requested at any available timepoint, including baseline, end of intervention, and any follow-up point. IPD was shared according to DTA conditions and stored securely in an access-controlled folder on the lead institution’s research drive.

L.K. and P.E.C. independently extracted relevant study-level data from included publications, using an adapted Cochrane data extraction form that was previously piloted within the review team (Supplementary Section 5.0).^55^ A third reviewer (J.M.) cross-checked extractions for accuracy. We asked original study authors to cross-check extractions and make amendments, if needed.

#### Data harmonization and checking

We harmonized data according to the prespecified data dictionary. We recoded and transformed IPD following a consistent coding scheme. As such, height and weight were transformed into metric units, age was converted into years, and EBTs were standardized by converting them to a common scale ranging from 0 to 100, with 0 indicating the lowest possible score and 100 the highest possible score. This was achieved by subtracting the raw outcome score (multiplied by the number of items contained within the subscale) by the lowest possible raw score, dividing this by the possible score range and multiplying this by 100. Harmonized IPD was merged into a combined dataset for analysis.

We implemented data checking procedures in 3 phases. Firstly, where item-level questionnaire data were provided, we recomputed EBT subscale scores to compare them with author-computed ones. Secondly, we compared descriptive data from the IPD of each trial to published reports. Thirdly, we checked descriptives before and after integrating the IPD into the combined dataset to confirm that the merging process did not introduce error. We discussed any discrepancies with trial authors. In cases where we could not resolve discrepancies, we assessed their severity among the study team. Trials with deviations were marked in the risk of bias assessment and excluded in sensitivity analyses (see “Risk of bias assessment”). Data cleaning, harmonizing, merging, and checking were performed using Stata v17.^56^

#### Studies where IPD is not available

We excluded trials that did not contribute IPD. Reasons for non-provision of IPD are summarised in Supplementary Table 6-1. For comparison purposes, we extracted their published data on sociodemographic details, EBTs, and weight loss outcomes in duplicate using a prespecified data extraction form. We then summarized their study characteristics in Supplementary Table 8-2 for comparison with included trials and provided their risk of bias rating in Supplementary Figure 7-2.

### Risk of bias assessment

Two reviewers, either L.K., P.E.C., or J.M., independently assessed the risk of bias of included trials using the Cochrane Risk of Bias tool 2 (RoB2).^57^ We resolved disagreements through discussion, and consulted a third reviewer, A.A., if necessary. Where information was missing from trial publications or for the assessment of ongoing studies, we asked authors directly. Given the differences between IPD and traditional aggregate meta-analyses, we adapted the ROB2 tool. We omitted domain 5 (selection of reported results), and we introduced an additional domain to reflect data discrepancies identified during the data checking process (see “Data harmonization and checking”). If data discrepancies were present for only some EBTs, we applied the risk of bias classification only to the EBT in question.

To address potential biases from the overall body of evidence, we compared study characteristics, outcomes, and risk of bias evaluations between trials with and without available IPD (see “Studies where IPD is not available”). We also investigated the risk of publication bias through contour enhanced funnel plots.

### Data analyses

#### Descriptive statistics

We calculated descriptive statistics for each trial separating intervention and control groups directly from IPD. We extracted additional characteristics, for which IPD was not available, from published reports.

#### Statistical analysis

To investigate the mediating effects of EBTs on weight outcomes in ACT-based interventions, we used a 1-stage IPD meta-analysis approach as described by Huh et al.^58^ This approach uses structural equation modeling to estimate (a) the direct effect of intervention group allocation on percent weight change from baseline to follow-up, (b) the indirect effect of the intervention group allocation on percent weight change from baseline to follow-up through changes in EBTs during the intervention period, and (c) the total effect, combining both the direct and indirect effect.

Each EBT was examined as a potential mediator in separate models. Each model controlled for the baseline EBT in question, baseline weight, sex/gender, time to intervention end, and time to follow-up. We used 2 sets of models, one for short-term and one for long-term mediating effects. For both models, changes in EBTs were calculated as change from baseline to the end of intervention. Percentage weight change in the short-term models was calculated as change from baseline to the follow-up closest to the end of intervention, typically around 6 months, and as change from baseline to exclusively 12 months after intervention end in the long-term models. Model follow-up times may have overlapped if no timepoint closer to the end of intervention than 12 months was available. We focused on investigating those EBTs that initially had more than 5 studies contributing data to them.

Estimates and confidence intervals were calculated using bootstrap resampling of 5000 bootstrapped datasets, and missing data were imputed using multiple imputation. We accounted for clustering within individual studies with a design-based approach by using complex survey analysis that applies weights to participants.^58^ The data were weighted using the inverse of the square root of each trial’s sample size, as outlined in Huh et al.^58^, to account for different sample sizes between trials.

We conducted analyses in the pooled sample, producing the “overall model,” as well as in “study-specific submodels” to illustrate heterogeneity in effects across studies. Study-specific submodels were performed in subsets of each individual study while basing imputation on data across all studies. Analyses were performed in R,^59^ adapting code published by Huh et al.^58^ that uses the lavaan^60^ and lavaan.survey^61^ packages.

#### Sensitivity analyses

We conducted a series of prespecified sensitivity analyses where more than 500 observations were available to accommodate the complexity of mediation models.^62^ At the study level, we set out to compare results from analyses performed in the full set of trials vs (a) trials classified as low risk of bias, (b) trials with minimal control conditions, (c) trials with standard behavioral control conditions, (d) trials that significantly reduced experiential avoidance, the target mechanism of action of ACT, and (e) trials measuring EBTs with the same questionnaire. At the individual level, we compared results from analyses performed in all individuals vs (f) those that received a sufficient dose of the intervention, considered to be equal to an attendance of at least 60% of sessions. Trials that did not assess variables of interest were not considered for sensitivity analyses.

### Patient and Public Involvement

Two Patient and Public Involvement (PPI) members with lived experience of obesity, dysfunctional eating behaviors, and weight management reviewed the findings and met with L.K. to discuss their implications. Insights generated in those meetings were incorporated into the discussion of findings.

## Results

### Study selection and characteristics

**Figure 1.**
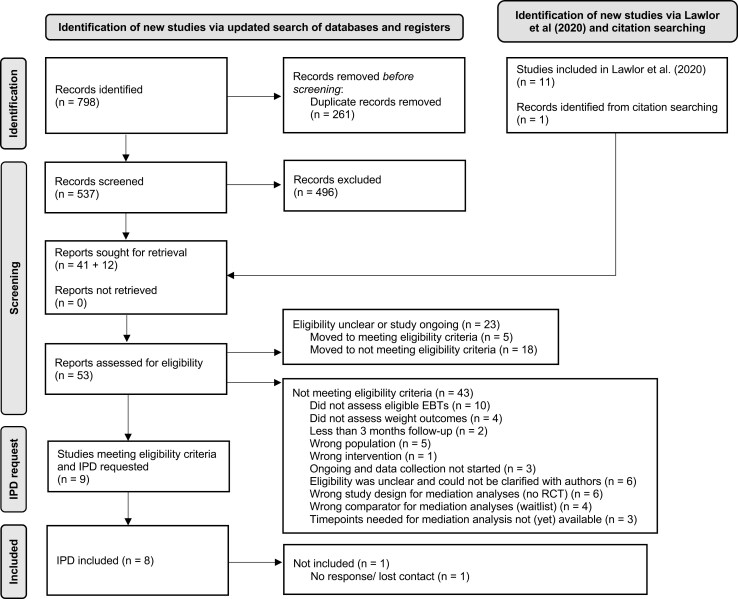
Flowchart depicting the study selection process.

We received IPD from 8 of 9 eligible trials, comprising 1391 observations. We were unable to obtain IPD from one trial due to losing contact with authors.^63^ The study selection process is illustrated in Figure 1. Table 1 provides an overview of study characteristics, with details on individual studies and intervention characteristics described in the Supplementary Table 8-1. Participant characteristics are summarized in Supplementary Tables 10-1–10-3.

**Table 1.** Overview of characteristics of included trials.

Study characteristics	*N* studies	Citations
Trial status		
Completed	5	[64–68]
Ongoing	3	[69–71]
Overall risk of bias rating		
Low	6^a^	[64–68,71]
Some concerns	2	[69,70]
High	0	n/a
Study location		
United States of America	7	[64–69,71]
Spain	1	[70]
Comparison type^b^		
Gold Standard (behavioral)	7	[64–69,71]
Usual care	1	[70]
Comparison intensity		
Low	0	n/a
High	7	[64–69,71]
Unknown^c^	1	[70]
Intervention duration		
≤12 weeks	0	n/a
12-26 weeks	1	[70]
≥26 weeks	7	[64–69,71]
Intervention delivery mode		
Face to face	6	[65–68,70,71]
Remote	0	n/a
Mixed	2	[64,69]
Intervention delivery format		
Individual	0	n/a
Group	5	[65,66,68,70,71]
Mixed	3	[64,67,69]
EBT questionnaire used^d^		
TFEQ-51	6	[64–69]
TFEQ-R18 or TFEQ-R21	2	[65,67]
DEBQ	2	[66,70]
EOQ	1	[64]
EES	2	[66,71]

*Abbreviations:* DEBQ, Dutch Eating Behaviour Questionnaire; EBT, eating behavior trait; EES, Emotional Eating Scale; EOQ, Emotional Overeating Questionnaire; *N* studies, number of studies; n/a, not applicable; TFEQ, Three-Factor Eating Questionnaire.

^a^Butryn [64] was classified as some concerns only for the emotional eating outcome due to data discrepancies.

^b^Multiple categories possible (eg, usual care and waitlist).

^c^Comparison intensity may be unknown for participants in usual care groups where healthcare support is provided and may depend on individuals’ insurance etc.

^d^Questionnaires might not have been administered in full, but only for select EBT subscales.

Most trials were based in the United States (*n* = 7), with one trial conducted in Spain (*n* = 1). Sample sizes ranged from 68^69^ to 317.^64^ The majority of participants were female (78%), with ages spanning from 18 to 71 years with a mean (SD) of 51 (10.94). The mean (SD) BMI was 36 kg/m^2^ (5.27), with a range from 26 to 64 kg/m^2^.

The mean (SD) duration of ACT-based interventions was 51 (18.29) weeks, varying from 20 to 78 weeks. Most interventions were conducted in face-to-face group settings (*n* = 5), while more recent trials included remote elements delivered to the individual (*n* = 3). Except for one trial, all compared ACT to active standard behavioral control conditions (*n* = 7). Most trials used the Three-Factor Eating Questionnaire (TFEQ) to assess EBTs (*n* = 6).

### IPD integrity

We identified no major data discrepancies during data checking. However, EBTs were often not reported in original publications, making it impossible to compare IPD against published data. Equally, for ongoing trials, no publications were yet available. One trial had noticeably lower emotional eating scores compared to others. Although we harmonized all EBT questionnaires onto a scale from 0 to 100, this deviance may stem from the use of a different questionnaire to assess emotional eating in this trial.^64^ This deviation was captured in the risk of bias assessment (Supplementary Figure 7-1).

We excluded observations that original trial authors marked as excluded. We also excluded observations containing biologically implausible values, as predefined in the analysis plan. Supplementary Table 9-1 summarizes excluded observations and reasons for exclusion.

### Risk of bias within and across studies

Most trials were rated as having low risk of bias (*n* = 6), while 2 trials raised some concerns due to baseline imbalances in weight (*n* = 1) or EBTs (*n* = 1) (see Supplementary Figure 7-1). One trial raised concerns only regarding the emotional eating outcome due to some minor discrepancies identified during data checking.

Overall, we deemed the risk of bias across studies to be low, as IPD was missing for only one eligible trial, and we included both published and ongoing/unpublished trials in this study. The excluded trial was conducted in US veterans, finding that the active standard BWMI control condition reduced weight significantly more than the ACT intervention did. However, a low sample size (*n* = 88) meant that the trial could have been underpowered.^63^ Four out of 8 included trials were conducted by the same research group,^64–67^ potentially increasing their similarity compared to the other trials. Funnel plots did not suggest evidence of publication bias; however, their interpretability was limited due to the low numbers of contributing trials (Supplementary Figures 13-1 and 13-2).^72,73^

### Mediating effects of EBTs on percentage weight change

**Figure 2.**
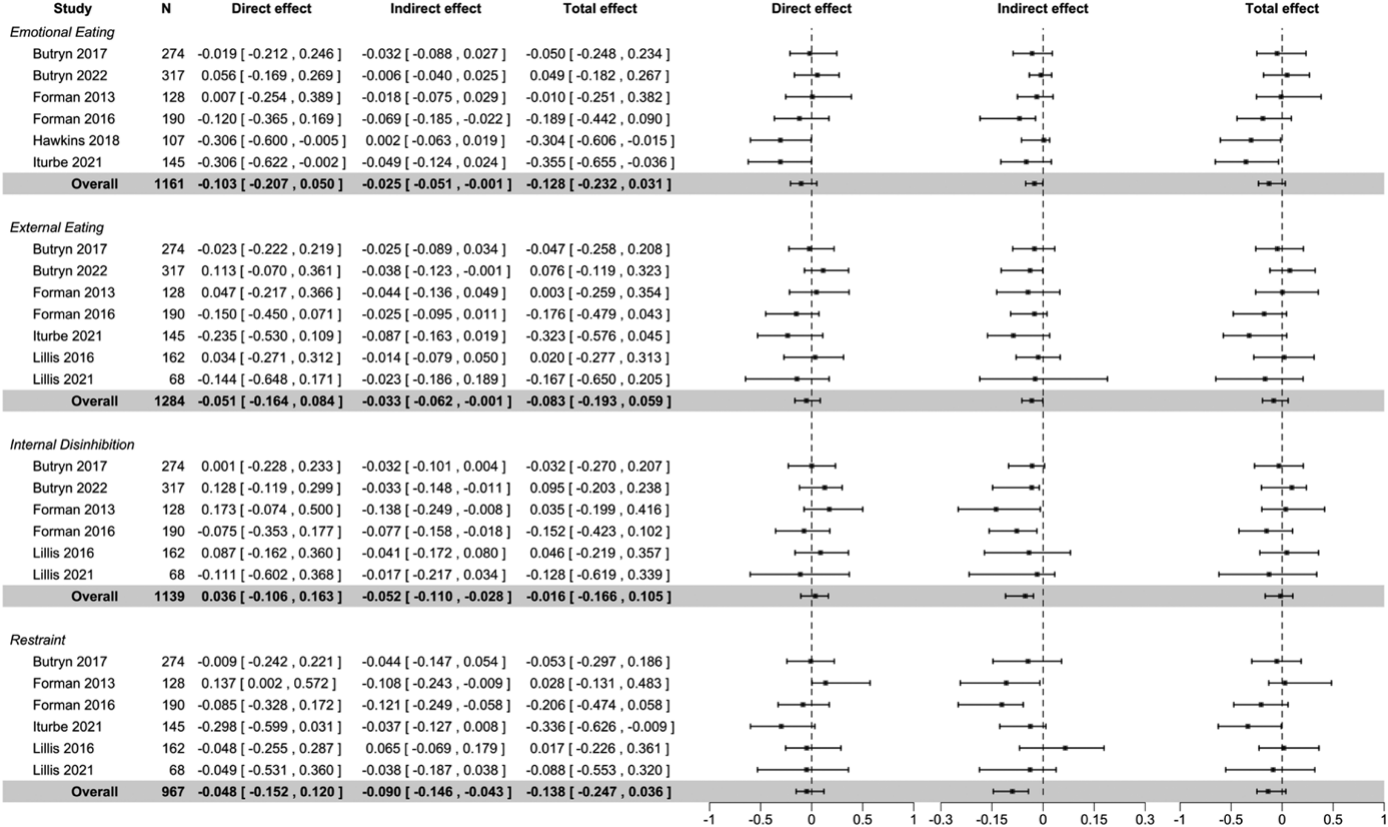
Forest plot of analyses exploring short-term mediating effects of ACT-based interventions through changes in EBTs. *Note:* Direct, indirect, and total effects are displayed as standardized mean difference [95% confidence interval] in percent weight change between intervention and control. The overall model results are highlighted in gray, while the study-specific submodels illustrate the heterogeneity of effects. Abbreviations: ACT, Acceptance and Commitment Therapy; EBT, eating behavior trait.

**Figure 3.**
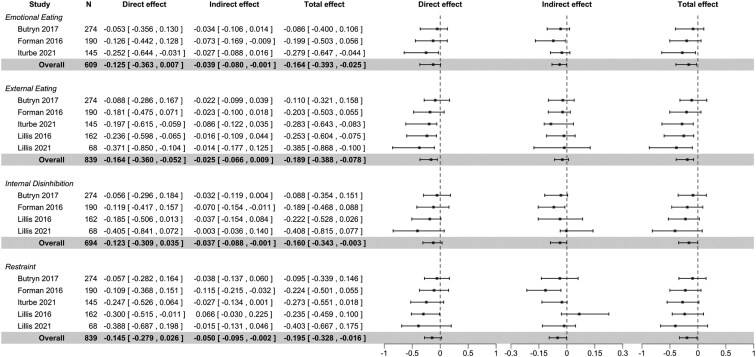
Forest plot of analyses exploring long-term mediating effects of ACT-based interventions through changes in EBTs. *Note:* Direct, indirect, and total effects are displayed as standardized mean difference [95% confidence interval] in percent weight change between intervention and control. The overall model results are highlighted in gray, while the study-specific submodels illustrate the heterogeneity of effects. Abbreviations: ACT, Acceptance and Commitment Therapy; EBT, eating behavior trait.

Four of the eligible EBTs initially had 5 or more contributing studies and were investigated for mediating effects (emotional eating, external eating, internal disinhibition, restraint). Due to their similarity, we combined external eating and external disinhibition into one trait. Estimates of the effect of randomized group on changes in EBTs are summarized in Supplementary Tables 1.1 and 1.2 for the samples used in short- and long-term analyses, respectively. Results from mediation analyses are summarized in Figure 2 for short-term effects and in Figure 3 for long-term effects. Path diagrams for each model are provided in Supplementary Figures 12-1–12-8.

We found evidence of a small effect of the intervention on changes from baseline to the end of intervention in external eating (−0.12 [95% CI, −0.23 to −0.01], *n* = 7), internal disinhibition (−0.16 [95% CI, −0.30 to −0.08], *n* = 6), and restraint (0.18 [95% CI, 0.09–0.28], *n* = 6), but not in emotional eating (−0.11 [95% CI, −0.21 to 0.01]) in overall models of the sample of studies contributing to the investigation of shorter-term effects (Supplementary Table 12-1). When using the sample of studies contributing to long-term effects, we only found a small effect of the intervention on changes in internal disinhibition (−0.14 [95% CI, −0.27 to −0.01], *n* = 4) and restraint (0.13 [95% CI, 0.0004–0.22], *n* = 5) from baseline to the end of intervention (Supplementary Table 12-2).

In overall models examining short-term mediating effects, we found no evidence of a direct or total effect of the intervention on percent weight change (Figure 2). However, we found significant indirect effects of the intervention on percentage weight change through changes in emotional eating (−0.02 [95% CI, −0.05 to −.001]), external eating (−0.03 [95% CI, −0.06 to −0.001]), internal disinhibition (−0.05 [95% CI, −0.11 to −0.02]), and restraint (−0.09 [95% CI, −0.14 to −0.04]) (Figure 2). Estimates are interpreted as the effect that a 1-unit change in the EBTs (assessed from a scale of 0 to 100) during the intervention period has on percentage weight change at the follow-up closest to the end of intervention, keeping all covariates in the model constant. For example, a 6-point reduction in internal disinhibition (reflecting the observed mean difference in change between the intervention and control groups [Supplementary Table 10-3]) would correspond to a −0.30% greater weight change in the ACT-based intervention compared to the control group, solely due to changes in internal disinhibition. For an individual with an initial weight of 120 kg, this translates to an additional 0.36 kg reduction attributable to this pathway. Study-specific submodels displayed some heterogeneity between studies, in particular for direct and total effects and for restraint (Figure 2).

When examining long-term mediating effects in overall models, we found evidence of both indirect and total effects of emotional eating (indirect: −0.03 [95% CI, −0.08 to −0.001]; total: −0.16 [95% CI, −0.39 to −0.02]), internal disinhibition (indirect: −0.03 [95% CI, −0.08 to −0.001]; total: −0.16 [95% CI, −0.34 to −0.003]), and restraint (indirect: −0.05 [95% CI, −0.09 to −0.002]; total: −0.19 [95% CI, −0.32 to −0.01]) on percent weight change, where the indirect effects explained 23.78%, 23.12%, and 25.64% of the total effects, respectively. For external eating, we found evidence of a direct (−0.16 [95% CI, −0.36 to −0.05]) and total effect (−0.19 [95% CI, −0.39 to −0.08]), but no evidence of an indirect effect (Figure 3). Again, study-specific submodels showed some heterogeneity between studies, although variability in direct and total effects appeared less compared to that in short-term models (Figure 3).

#### Sensitivity analyses

An overview of the sensitivity analyses results for the overall models investigating short- and long-term effects is provided in Supplementary Tables 14-1 and 14-2. Overall, the direction of indirect and total effect estimates was consistent across all sensitivity analyses of all EBTs, favoring a total reduction in percentage bodyweight and a reduction in weight through changes in EBT. We found no evidence of direct effects for any sensitivity analyses and EBTs, and the direction of estimates varied.

For short-term analyses of emotional eating, we found evidence of an indirect, but no evidence of a total effect in trials with standard behavioral comparisons. In trials with low risk of bias, we found evidence of a total effect but no evidence of an indirect effect. In participants with at least 60% attendance, we found evidence for both an indirect and total effect, with a proportion of 21.0% of the total effect of the intervention on weight change being explained by changes in emotional eating. An insufficient number of observations meant that we did not conduct sensitivity analyses for long-term mediating effects of emotional eating.

In short-term analyses of external eating, we found evidence of an indirect effect and total effect in participants with at least 60% attendance, with 31.3% of the total effect explained by changes in external eating. There was no evidence of an indirect effect in the remaining short- and long-term sensitivity analyses.

For internal disinhibition, we consistently found evidence of a significant indirect effect favoring weight loss across all short-term sensitivity analyses, without evidence of a total effect. The strongest effects were observed in trials that reduced experiential avoidance and in participants with at least 60% attendance. In the long-term sensitivity analysis of trials with a standard behavioral comparison, there was evidence of both an indirect and total effect, with 23.12% if the total effect on weight change being explained through changes in internal disinhibition. No further long-term sensitivity analyses were conducted due to an insufficient number of observations.

Finally, in short-term analyses of restraint, we found evidence of an indirect, but no evidence of a total effect in trials with standard behavioral comparisons and those that significantly reduced experiential avoidance. In sensitivity analyses of participants with at least 60% attendance, we found evidence for both an indirect and total effect, with 55.30% of the total effect on weight change explained by changes in restraint. In the long-term analysis of trials with a standard behavioral comparison, we found evidence of a total, but not an indirect effect. We were unable to conduct additional long-term sensitivity analyses.

## Discussion

To our knowledge, this IPD meta-analysis is the first study to investigate EBTs as mechanisms through which ACT-based BWMIs facilitate weight loss. Overall, we found participants in ACT interventions lost an average of 7.36% of their initial body weight in the short-term (calculated as change from baseline to the follow-up closest to the end of intervention, typically around 6 months) and 6.65% in the longer-term (calculated as change from baseline to exclusively 12 months after intervention end). Our analyses suggest small partial mediating effects of ACT-based interventions on short-term weight loss through changes in emotional eating, external eating, internal disinhibition and restraint, and on long-term weight loss through changes in emotional eating, internal disinhibition, and restraint.

Mediating effects were inferred based on the presence of significant indirect effects of the interventions on weight loss via changes in these EBTs and an absence of evidence for significant direct intervention effects in the same models. However, in the short term, we also found no evidence to suggest total effects of the interventions on weight change. This may be explained by the small indirect effect sizes and the greater variability of total effects that require more statistical power to detect an effect.^74–76^ The absence of evidence for total effects necessitates caution when interpreting mediating effects in the short term. On the other hand, we did find evidence for the presence of total effects in the long term, with the indirect effects accounting for up to 25.64% of the overall intervention impact on weight change. This indicates that while certain EBTs are suggested to be mechanisms through which ACT-based interventions influence weight loss, other unmeasured mediators may also be influencing the total effect, and the mediating impact of EBTs can be considered partial.^77,78^ Future research should identify unmeasured mediators, which may include target mechanisms of ACT-based BWMIs such as psychological flexibility and experiential avoidance,^43^ and compare them with EBTs in combined mediation models to further disentangle their effects.

Consistent with our findings, previous studies have also identified significant mediating effects of restraint,^35,38,39^ emotional eating,^35^ and disinhibition^35–37^ in a variety of intervention types. Only one study found no mediating effects of emotional eating,^79^ possibly due to their low sample size (*n* = 39). In our analyses, changes in EBTs explained 23%-26% of the total intervention effect on weight change. This proportion is higher than the ~5% mediation through disinhibition observed in structured meal- or nutrition-based interventions without psychological focus by JaKa et al.^36^ and Dorling et al.,^37^ but lower than the 50%-80% mediation seen for other psychological mechanisms (eg, distress, quality of life, weight stigma) in a small (*n* = 84) study of an ACT-based BWMI,^80^ and the ~55% mediation through restraint in a small (*n* = 98) intensive nutrition-based intervention.^39^ Additionally, in absolute terms, the mediating effects identified in this IPD meta-analysis were small, with a 1-unit change in EBTs corresponding to a −0.02 to −0.09 percent weight change through the indirect path. Furthermore, study-specific submodels displayed some heterogeneity across trials, particularly in the short term. Thus, although our findings provide initial evidence of a partial mediating role of EBTs in ACT-based BWMIs in both the short and long term, large confirmatory trials are required before applying findings to practice.

Several aspects may contribute to the modest size of mediating effects. Notably, the majority of trials in our sample (7 out of 8) used active standard behavioral interventions as control conditions, likely making it more challenging to detect differences between groups. Furthermore, the effects of the ACT-based interventions on changes in EBTs were small, including small reductions in external eating (−0.12 [95% CI, −0.23 to −0.01]) and internal disinhibition (−0.16 [95% CI, −0.3 to −0.08]), a small increase in restraint (0.18 [95% CI, 0.09–0.28]), and no evidence of an intervention effect on emotional eating. The magnitude of mediating effects may also differ according to baseline levels of EBTs.^81^ Previous aggregate meta-analyses of ACT-based BWMIs and other third-wave cognitive behavioral interventions have also found changes in EBTs like emotional eating,^45^ external eating,^82^ restraint,^46^ and general disinhibition.^46^ However, some found no evidence of an effect on some EBTs.^46,82^

These mixed findings on the impact of ACT-based interventions on EBTs may be explained by heterogeneity in the included studies relating to the development and content of ACT-based interventions. ACT generally builds on 6 core components (ie, acceptance, cognitive defusion, self as context, present moment awareness, values, and committed action) ^43,83^ but is not implemented consistently across interventions. It is currently unclear which intervention components may lead to changes in which EBT.^84,85^ Additionally, people may display a combination of different EBTs, requiring treatment that uses multiple approaches or components. Future research should explore ways to refine BWMIs to more effectively target EBTs. This may include investigating the impact of different ACT components on EBTs but also considering other targeted intervention types^86^ and intervention characteristics, such as interventionists’ expertise^66^ or intervention duration,^87,88^ and exploring approaches to address a combination of distinct EBTs.

Sensitivity analyses may provide additional insights into understanding the context of mediating effects. For example, in a subset of participants that were considered to have received a sufficient dose of the intervention (≥60% attendance), we found effect sizes of indirect effects of all EBTs were larger. There were total effects for emotional eating, external eating, and restraint in the short term, leading to 21.0%, 31.3%, and 55.30% of the total effect being explained by changes in the respective EBT. This may provide further confidence in our findings and indicate that, when administered as intended, ACT-based interventions are initially likely to work, in-part, through changing EBTs.

We also conducted sensitivity analyses in trials with an active standard BWMI comparison group to compare working mechanisms of these different intervention types. Findings suggest that in comparison to standard BWMIs, ACT-based interventions had a greater impact on weight loss outcomes through changes in emotional eating, external eating, internal disinhibition, and restraint in the short term. In the long term, we replicated the mediating effect of internal disinhibition observed in the main analyses but did not find evidence of a mediating effect for restraint. This could be due to ACT not explicitly targeting restraint as a mechanism of change.^43^ Thus, while ACT may initially work to reduce weight more strongly than standard BWMIs through changing a variety of EBTs, including restraint, over time, other mediators like internal disinhibition may play a more prominent role in ACT-based interventions. Future studies are needed to replicate these initial observations and compare the role of EBTs across other intervention types that aim to target different EBTs.

### Strengths and limitations

A major strength of this study was the use of IPD identified through a systematic search across 8 databases. This enabled us to compile a large database including published and unpublished trials. Using a 1-stage analysis increased statistical power compared to individual trials and conventional meta-analyses. Multiple imputation of missing data and the provision of bootstrapped confidence intervals produced more robust effect estimates.

Methodological constraints and limitations related to the included sample should be considered when interpreting findings. All except one included trial were conducted in the United States, and most participants were women (78%), which may limit the generalizability of findings to other populations. Additionally, all except one included trial had an active standard BWMI comparison. As described in the discussion, comparing ACT to mostly high-intensity active control groups could have contributed to difficulties identifying a significant total difference between groups and may have led to smaller effect sizes. On the other hand, it could be considered promising to have identified mediating effects of ACT with mostly active, high-intensity comparison groups. Not all included trials measured all eligible EBTs, and sample sizes were further reduced when selecting subsamples for sensitivity analyses. This meant that we could not perform all planned sensitivity analyses, particularly in the long-term period, or estimate mediating effects of EBTs in a combined model. This is of particular relevance to compare the role of different EBTs in ACT-based interventions and determine the extent to which their effects are independent from one another. Finally, while utilizing RCTs enabled us to assume unconfounded intervention–mediator and intervention–outcome relationships, there may be confounding for the mediator–outcome path.^77,89–91^ We cannot rule out confounding by unmeasured variables, such as the expertise of those delivering the intervention.^66^

### Directions for future research

Given the initial evidence on mediating effects of EBTs and the small effects of the included interventions on changes in EBTs, future research may wish to investigate ways to better address these traits. This could include disentangling which intervention components have the greatest impact on target traits, utilizing innovative intervention designs such as those guided by the MOST framework.^84,92,93^ It may also be relevant to investigate the impact of other intervention types and characteristics, including aspects surrounding the intervention development and delivery, on changes in EBTs. Qualitative research and stakeholder involvement in intervention development may provide particularly valuable insights. Future trials should also aim to assess EBTs consistently and include longer follow-ups for further exploration of long-term effects. These trials may also assess the mediating role of EBTs alongside other potential mediators of ACT-based BWMIs, such as psychological flexibility, experiential avoidance, and other personal characteristics, allowing for a direct comparison of their relative contributions to weight loss and further informing their clinical relevance as intervention targets. Lastly, data sharing should be facilitated to enable easier implementation of IPD meta-analyses that is needed to investigate mediators and moderators with increased statistical power.^81,94^

## Conclusion

The effect of ACT-based interventions on weight loss was, in-part, mediated by changes in emotional eating, external eating, internal disinhibition, and restraint in the short term, and by changes in emotional eating, internal disinhibition, and restraint in the long term. However, mediating effects were small, as were the effects of the intervention on changing EBTs. Future research should identify more effective strategies to target different EBTs.

## Supplementary Material

kaaf039_suppl_Supplementary_Sections

## Transparency statement

Ethical approval was obtained from the Cambridge Psychology Research Ethics Committee (Application No: PRE.2023.121). The project was preregistered on PROSPERO (CRD42022359691), with a study protocol published in *BMJ Open*.^50^ We also submitted an analysis plan to the Open Science Forum (OSF) prior to commencing analyses (doi: 10.17605/OSF.IO/3X6YM). Data for this IPD meta-analysis were shared by trialists under data sharing agreements that do not allow for onward sharing. Requests for participant-level data should be made to the original owners of the data. Analytic code and study materials may be available by emailing the corresponding author.

## Rights retention statement

For the purpose of Open Access, the author has applied a Creative Commons Attribution (CC BY) licence to any Author Accepted Manuscript version arising.
