# Supplementary material for: An individual participant data meta-analysis investigating the mediating role of eating behavior traits in Acceptance and Commitment Therapy-based weight management interventions
Source: Ann Behav Med. 2025 Jun 2;59(1):kaaf039. doi: 10.1093/abm/kaaf039 (PMC12169333; doi:10.1093/abm/kaaf039)
Supplement: kaaf039_suppl_Supplementary_Sections [file kaaf039_suppl_supplementary_sections.pdf]

An Individual Participant Data meta-analysis investigating the mediating role of Eating Behaviour Traits in Acceptance and Commitment Therapy-based weight management interventions

[SUPPLEMENTARY MATERIAL]

Table of Contents

1.0 PRISMA-IPD Checklist 4
2.0 AGReMA Checklist 8
3.0 PICO(S) terms 11
4.0 Full electronic search strategy 12
4.1 MEDLINE via Ovid 12
4.2 EMBASE via Ovid 12
4.3 CENTRAL via Cochrane 12
4.4 ASSIA via ProQuest 14
4.5 WEB OF SCIENCE via web of science 14
4.6 CINAHL via EBSCOhost 14
4.7 AMED via Ovid 14
4.8 PSYCINFO via ProQuest 15
5.0 Data extraction form 16
6.0 Reasons for non-provision of IPD 19
7.0 Risk of Bias assessments 20
7.1 RoB assessment of studies providing IPD 20
7.2 RoB assessment of studies not providing IPD 20
8.0 Study characteristics 21
8.1 Study characteristics of studies providing IPD 21
8.2 Study characteristics of studies not providing IPD 39
9.0 Number of excluded observations and reasons 41
10.0 Descriptive participant characteristics 42
11.0 Intervention effects on changes in EBTs 45
12.0 Path diagrams 47
13.0 Contour-enhanced funnel plots of indirect effects 51
14.0 Sensitivity analyses of short- and long-term mediation models 53
15.0 References Supplementary Material 55

Tables

Table SM 1-1: PRISMA-IPD Checklist indicating the manuscripts fulfillment of outlined criteria 4
Table SM 2-1: AGReMA Checklist indicating the manuscripts fulfillment of outlined criteria 8
Table SM 3-1: PICO(S) terms indicating eligibility criteria guiding the identification and inclusion of studies 11
Table SM 6-1: Reasons for not providing IPD and exclusion 19
Table SM 8-1: Detailed characteristics of included studies that provided IPD as extracted from published manuscripts 21
Table SM 9-1: Number of excluded observations and reasons for exclusion as indicated by original study authors 41
Table SM 10-1*:* Participant characteristics (sex, age, baseline weight) of included studies as derived from individual participant data 42
Table SM 10-2*:* Participant weight change in included studies as derived from individual participant data 43
Table SM 10-3: Participant changes in eating behaviour traits in included studies as derived from individual participant data 44
Table SM 11-1: Intervention effects on changes in EBTs with short-term sample 45
Table SM 11-2: Intervention effects on changes in EBTs with long-term sample 46
Table SM 14-1: Overview of sensitivity analyses displaying the overall direct, indirect and total effects of short-term mediating models 53
Table SM 14-2: Overview of sensitivity analyses displaying the overall direct, indirect and total effects of long-term mediating models 54

Figures

Figure SM 7-1: Risk of Bias assessment of included studies using a modified version of the Cochrane Risk of Bias tool 2 (RoB2) 20
Figure SM 7-2: Risk of Bias assessment of eligible studies that did not provide IPD using a modified version of the Cochrane Risk of Bias tool 2 (RoB2) 20
Figure SM 12-1: Path diagram for short-term emotional eating model 47
Figure SM 12-2: Path diagram for long-term emotional eating model 47
Figure SM 12-3: Path diagram for short-term external eating model 48
Figure SM 12-4: Path diagram for long-term external eating model 48
Figure SM 12-5: Path diagram for short-term internal disinhibition model 49
Figure SM 12-6: Path diagram for long-term internal disinhibition model 49
Figure SM 12-7: Path diagram for short-term restraint model 50
Figure SM 12-8: Path diagram for long-term restraint model 50
Figure SM 13-1: Contour-enhanced funnel plots of indirect effects on percentage weight change through changes in eating behaviour traits in short-term mediation models 51
Figure SM 13-2: Contour-enhanced funnel plots of indirect effects on percentage weight change through changes in eating behaviour traits in long-term mediation models 52

1.0 PRISMA-IPD Checklist

Table SM 1-1: PRISMA-IPD Checklist indicating the manuscripts fulfillment of outlined criteria

PRISMA-IPD Section/topic	Item No	Checklist item	**Reported on page**
Title			
Title	1	Identify the report as a systematic review and meta-analysis of individual participant data.	1
Abstract			
Structured summary	2	Provide a structured summary including as applicable:	1
		**Background**: state research question and main objectives, with information on participants, interventions, comparators and outcomes.	
		**Methods**: report eligibility criteria; data sources including dates of last bibliographic search or elicitation, noting that IPD were sought; methods of assessing risk of bias.	
		**Results**: provide number and type of studies and participants identified and number (%) obtained; summary effect estimates for main outcomes (benefits and harms) with confidence intervals and measures of statistical heterogeneity. Describe the direction and size of summary effects in terms meaningful to those who would put findings into practice.	
		**Discussion:** state main strengths and limitations of the evidence, general interpretation of the results and any important implications.	
		**Other:** report primary funding source, registration number and registry name for the systematic review and IPD meta-analysis.	
Introduction			
Rationale	3	Describe the rationale for the review in the context of what is already known.	3-4
Objectives	4	Provide an explicit statement of the questions being addressed with reference, as applicable, to participants, interventions, comparisons, outcomes and study design (PICOS). Include any hypotheses that relate to particular types of participant-level subgroups.	5
Methods			
Protocol and registration	5	Indicate if a protocol exists and where it can be accessed. If available, provide registration information including registration number and registry name. Provide publication details, if applicable.	(Title page submitted separately from the manuscript)
Eligibility criteria	6	Specify inclusion and exclusion criteria including those relating to participants, interventions, comparisons, outcomes, study design and characteristics (e.g. years when conducted, required minimum follow-up). Note whether these were applied at the study or individual level i.e. whether eligible participants were included (and ineligible participants excluded) from a study that included a wider population than specified by the review inclusion criteria. The rationale for criteria should be stated.	5

Identifying studies - information sources	7	Describe all methods of identifying published and unpublished studies including, as applicable: which bibliographic databases were searched with dates of coverage; details of any hand searching including of conference proceedings; use of study registers and agency or company databases; contact with the original research team and experts in the field; open adverts and surveys. Give the date of last search or elicitation.	5
Identifying studies - search	8	Present the full electronic search strategy for at least one database, including any limits used, such that it could be repeated.	SM 4.0
Study selection processes	9	State the process for determining which studies were eligible for inclusion.	6
Data collection processes	10	Describe how IPD were requested, collected and managed, including any processes for querying and confirming data with investigators. If IPD were not sought from any eligible study, the reason for this should be stated (for each such study).	6-7
		If applicable, describe how any studies for which IPD were not available were dealt with. This should include whether, how and what aggregate data were sought or extracted from study reports and publications (such as extracting data independently in duplicate) and any processes for obtaining and confirming these data with investigators.	
Data items	11	Describe how the information and variables to be collected were chosen. List and define all study level and participant level data that were sought, including baseline and follow-up information. If applicable, describe methods of standardising or translating variables within the IPD datasets to ensure common scales or measurements across studies.	6
IPD integrity	A1	Describe what aspects of IPD were subject to data checking (such as sequence generation, data consistency and completeness, baseline imbalance) and how this was done.	6-7
Risk of bias assessment in individual studies.	12	Describe methods used to assess risk of bias in the individual studies and whether this was applied separately for each outcome. If applicable, describe how findings of IPD checking were used to inform the assessment. Report if and how risk of bias assessment was used in any data synthesis.	7-8
Specification of outcomes and effect measures	13	State all treatment comparisons of interests. State all outcomes addressed and define them in detail. State whether they were pre-specified for the review and, if applicable, whether they were primary/main or secondary/additional outcomes. Give the principal measures of effect (such as risk ratio, hazard ratio, difference in means) used for each outcome.	8-9
Synthesis methods	14	Describe the meta-analysis methods used to synthesise IPD. Specify any statistical methods and models used. Issues should include (but are not restricted to):<br>• Use of a one-stage or two-stage approach.<br>• How effect estimates were generated separately within each study and combined across studies (where applicable).<br>• Specification of one-stage models (where applicable) including how clustering of patients within studies was accounted for.<br>• Use of fixed or random effects models and any other model assumptions, such as proportional hazards.<br>• How (summary) survival curves were generated (where applicable).<br>• Methods for quantifying statistical heterogeneity (such as I^2 and τ^2).	8-9

		• How studies providing IPD and not providing IPD were analysed together (where applicable).<br>• How missing data within the IPD were dealt with (where applicable).	
Exploration of variation in effects	A2	If applicable, describe any methods used to explore variation in effects by study or participant level characteristics (such as estimation of interactions between effect and covariates). State all participant-level characteristics that were analysed as potential effect modifiers, and whether these were pre-specified.	9-10
Risk of bias across studies	15	Specify any assessment of risk of bias relating to the accumulated body of evidence, including any pertaining to not obtaining IPD for particular studies, outcomes or other variables.	7-8
Additional analyses	16	Describe methods of any additional analyses, including sensitivity analyses. State which of these were pre-specified.	9-10
Results			
Study selection and IPD obtained	17	Give numbers of studies screened, assessed for eligibility, and included in the systematic review with reasons for exclusions at each stage. Indicate the number of studies and participants for which IPD were sought and for which IPD were obtained. For those studies where IPD were not available, give the numbers of studies and participants for which aggregate data were available. Report reasons for non-availability of IPD. Include a flow diagram.	10-11
Study characteristics	18	For each study, present information on key study and participant characteristics (such as description of interventions, numbers of participants, demographic data, unavailability of outcomes, funding source, and if applicable duration of follow-up). Provide (main) citations for each study. Where applicable, also report similar study characteristics for any studies not providing IPD.	10-11
IPD integrity	A3	Report any important issues identified in checking IPD or state that there were none.	11
Risk of bias within studies	19	Present data on risk of bias assessments. If applicable, describe whether data checking led to the up-weighting or down-weighting of these assessments. Consider how any potential bias impacts on the robustness of meta-analysis conclusions.	11-12
Results of individual studies	20	For each comparison and for each main outcome (benefit or harm), for each individual study report the number of eligible participants for which data were obtained and show simple summary data for each intervention group (including, where applicable, the number of events), effect estimates and confidence intervals. These may be tabulated or included on a forest plot.	Not applicable to one-stage IPD
Results of syntheses	21	Present summary effects for each meta-analysis undertaken, including confidence intervals and measures of statistical heterogeneity. State whether the analysis was pre-specified, and report the numbers of studies and participants and, where applicable, the number of events on which it is based.	12-14
		When exploring variation in effects due to patient or study characteristics, present summary interaction estimates for each characteristic examined, including confidence intervals and measures of statistical heterogeneity. State whether the analysis was pre-specified. State whether any interaction is consistent across trials.	

		Provide a description of the direction and size of effect in terms meaningful to those who would put findings into practice.	
Risk of bias across studies	22	Present results of any assessment of risk of bias relating to the accumulated body of evidence, including any pertaining to the availability and representativeness of available studies, outcomes or other variables.	11-12
Additional analyses	23	Give results of any additional analyses (e.g. sensitivity analyses). If applicable, this should also include any analyses that incorporate aggregate data for studies that do not have IPD. If applicable, summarise the main meta-analysis results following the inclusion or exclusion of studies for which IPD were not available.	13-14
Discussion			
Summary of evidence	24	Summarise the main findings, including the strength of evidence for each main outcome.	15
Strengths and limitations	25	Discuss any important strengths and limitations of the evidence including the benefits of access to IPD and any limitations arising from IPD that were not available.	17-18
Conclusions	26	Provide a general interpretation of the findings in the context of other evidence.	19
Implications	A4	Consider relevance to key groups (such as policy makers, service providers and service users). Consider implications for future research.	18
Funding			
Funding	27	Describe sources of funding and other support (such as supply of IPD), and the role in the systematic review of those providing such support.	(Title page submitted separately from the manuscript)

A1 – A3 denote new items that are additional to standard PRISMA items. A4 has been created as a result of re-arranging content of the standard PRISMA statement to suit the way that systematic review IPD meta-analyses are reported.

© Reproduced with permission of the PRISMA IPD Group, which encourages sharing and reuse for non-commercial purposes

2.0 AGReMA Checklist

Table SM 2-1: AGReMA Checklist indicating the manuscripts fulfillment of outlined criteria

Section/Topic	Item Number	Item Description	Reported on page No
**Title and abstract**			
Title	1	Identify that the study uses mediation analysis	1
Abstract	2	Provide a structured summary of the objectives, methods, results, and conclusions specific to mediation analyses	1
**Introduction**			
Background and rationale	3	Describe the study background and theoretical rationale for investigating the mechanisms of interest. Include supporting evidence or theoretical rationale for why the intervention or exposure might have a causal relationship with the proposed mediators. Include supporting evidence or theoretical rationale for why the mediators might have a causal relationship with the outcomes	3-4
Objectives	4	State the objectives of the study specific to the mechanisms of interest. The objectives should specify whether the study aims to test or estimate the mechanistic effects	5
**Methods**			
Study registration	5	If applicable, provide references to any protocols or study registrations specific to the mediation analysis, and highlight any deviations from the planned protocol	(Title page submitted separately from the manuscript)
Study design and source of data	6	Specify the design of the original study that was used in mediation analyses and where the details can be accessed, supported by a reference. If applicable, describe study design features that are relevant to mediation analyses	5-7
Participants	7	Describe the target population, eligibility criteria specific to mediation analyses, study locations, and study dates (start of participant enrolment and end of follow-up)	5
Sample Size	8	State whether a sample size calculation was conducted for mediation analyses. If so, explain how it was calculated	Not applicable to IPD meta-analyses
Effects of interest	9	Specify the effects of interest	8-9
Assumed causal model	10	Include a graphic representation of the assumed causal model including the exposure, mediator, outcome, and possible confounders	Supplementary Material section 12
Causal assumptions	11	Specify assumptions about the causal model	8-9

Measurement	12	Clearly describe the interventions or exposures, mediators, outcomes, confounders, and moderators that were used in the analyses. Specify how and when they were measured, the measurement properties, and whether blinded assessment was used	6-7, 8-9
Measurement levels	13	If relevant, describe the levels at which the exposure, mediator, and outcome were measured	6-7
Statistical methods	14	Describe the statistical methods used to estimate the causal relationships of interest. This description should specify analytical strategies used to reduce confounding, model building procedures, justification for the inclusion or exclusion of possible interaction terms, modelling assumptions, and methods used to handle missing data. Provide a reference to the statistical software and package used	8-9
Sensitivity analyses	15	Describe any sensitivity analyses that were used to explore causal or statistical assumptions and the influence of missing data	9-10
Ethical approval	16	Name the institutional research board or ethics committee that approved the study. Provide a description of participant informed consent or ethics committee waiver of informed consent	(Title page submitted separately from the manuscript)
**Results**			
Participants	17	Describe baseline characteristics of participants included in mediation analyses. Report the total sample size and number of participants lost during follow-up or with missing data	10-11
Outcomes and estimates	18	Report point estimates and uncertainty estimates for the exposure-mediator and mediator-outcome relationships. If inference concerning the causal relationship of interest is considered feasible given the causal assumptions, report the point estimate and uncertainty estimate	12-13
Sensitivity parameters	19	Report the results from any sensitivity analyses used to assess robustness of the causal or statistical assumptions, and the influence of missing data	13-14
**Discussion**			
Limitations	20	Discuss the limitations of the study including potential sources of bias	17-18
Interpretation	21	Interpret the estimated effects considering the study’s magnitude and uncertainty, plausibility of the causal assumptions, limitations, generalizability of the findings, and results from relevant studies	15-17
Implications	22	Discuss the implications of the overall results for clinical practice, policy, and science	18-19
**Other information**			
Funding and role of sponsor	23	List all sources of funding or sponsorship for the mediation analysis and the role of the funders/sponsors in the conduct of the study, writing of the manuscript, and decision to submit for publication.	(Title page submitted separately

			from the manuscript)
Conflicts of interest and financial disclosures	24	State any conflicts of interest and financial disclosures for all authors	(Title page submitted separately from the manuscript)
Data and code	25	Authors are encouraged to provide a statement for sharing data and code for the mediation analysis	(Title page submitted separately from the manuscript)

From: Lee H, Cashin AG, Lamb SE, Hopewell S, Vansteelandt S, VanderWeele TJ, et al. A Guideline for Reporting Mediation Analyses of Randomized Trials and Observational Studies. The AGReMA Statement. JAMA. 2021;326(11):1045–1056. doi:10.1001/jama.2021.14075

3.0 PICO(S) terms

Table SM 3-1: PICO(S) terms indicating eligibility criteria guiding the identification and inclusion of studies

PICOs	Eligibility
Population	• Adults (aged 18 and older) with a BMI ≥ 25 kg/m2.<br>• Studies were excluded if participants were recruited purely based on having a chronic disease or being pregnant, as were studies where eligible participants resided in institutional settings (e.g. hospital, army barracks).<br>• Studies on children and adolescents were not considered for inclusion to avoid the risk of increasing heterogeneity in interventions serving different target populations.
Intervention	• Interventions with the primary goal of weight loss or weight loss maintenance (referred to as weight management interventions in the remainder of this protocol) that report incorporating strategies based on ACT.<br>• ACT-based interventions from different contexts were eligible (e.g. online, in person, health care setting, commercial), and they were eligible either as standalone treatment or as part of a wider weight management intervention.
Comparison	• Inactive control, minimal intervention (e.g. leaflet, brief advice), or an active standard behavioural weight management control
Outcomes	• Outcome: Weight assessed at both baseline and any follow-up post intervention end. Additionally, a follow-up point of at least 3-months post-baseline had to be available.<br>• Effect modifiers of interest: EBTs assessed at both baseline and intervention end. Eligible EBTs were emotional eating, external eating, disinhibition (general disinhibition, internal disinhibition, external disinhibition), restraint (general restraint, flexible restraint, rigid restraint) and uncontrolled eating.
Study Design	• RCTs and cluster-RCTs

4.0 Full electronic search strategy

4.1 MEDLINE via Ovid

exp Obesity/ OR exp Overweight/ OR exp Body Weight/ OR exp Body Mass Index/ OR exp Waist Circumference/ OR exp Feeding Behavior/ OR exp Body Weight Changes/ OR exp Caloric Restriction/ OR exp Weight Loss/ OR obes*.mp OR (overweight or over-weight).mp OR (weight adj3 (body or chang* or loss* or maint* or manag* or control* or reduct*)).mp OR (food adj3 (intake or habit*)).mp OR (body mass index or bmi).mp OR body adj3 mass.mp OR (calori* adj3 (restrict* or restrain* or reduc*)).mp OR feeding adj3 behavio*.mp OR (diet* adj3 (restrict* or restrain* or reduc*)).mp OR (waist* adj3 circumferenc*).mp

AND

"Acceptance and Commitment Therapy"/ OR (acceptance* adj3 (commit* or mind* or base* or focus* or intervention* or therap* or treat*)).mp

Limit NUM to dt=20190925-20220620

Limit NUM to rd=20190925-20220620

NUM or NUM

4.2 EMBASE via Ovid

exp Obesity/ OR exp Body Weight/ OR exp Body Mass/ OR exp Waist Circumference/ OR exp Feeding Behavior/ OR exp Caloric Restriction/ OR exp Weight Reduction/ OR obes*.mp OR (overweight or over-weight).mp OR (weight adj3 (body or chang* or loss* or maint* or manag* or control* or reduct*)).mp OR (food adj3 (intake or habit*)).mp OR (body mass index or bmi).mp OR (body adj3 mass).mp OR (calori* adj3 (restrict* or restrain* or reduc*)).mp OR (feeding adj3 behavio*).mp OR (diet* adj3 (restrict* or restrain* or reduc*)).mp OR (waist* adj3 circumferenc*).mp

AND

"acceptance and commitment therapy"/ OR (acceptance* adj3 (commit* or mind* or base* or focus* or intervention* or therap* or treat*)).mp

Limit NUM to dd=20190925-20220620

Limit NUM to rd=20190925-20220620

NUM or NUM

4.3 CENTRAL via Cochrane

#1	MeSH descriptor: [Obesity] explode all trees	15762

#2	MeSH descriptor: [Overweight] explode all trees	18878
#3	MeSH descriptor: [Body Weight] explode all trees	31138
#4	MeSH descriptor: [Body Mass Index] explode all trees	10927
#5	MeSH descriptor: [Waist Circumference] explode all trees	1143
#6	MeSH descriptor: [Feeding Behavior] explode all trees	9769
#7	MeSH descriptor: [Body Weight Changes] explode all trees	9695
#8	MeSH descriptor: [Caloric Restriction] explode all trees	941
#9	MeSH descriptor: [Weight Loss] explode all trees	7104
#10	obes* in All Text	51603
#11	(overweight or over-weight)	19265
#12	(weight near/3 (body or chang* or loss* or maint* or manag* or control* or reduct*))	75571
#13	(food near/3 (intake or habit*))	11473
#14	(body mass index or bmi)	74353
#15	(body near/3 mass)	64975
#16	(calori* near/3 (restrict* or restrain* or reduc*))	3305
#17	feeding near/3 behavio*	5662
#18	(diet* near/3 (restrict* or restrain* or reduc*))	15513
#19	(waist* near/3 circumferenc*)	10789
#20	#1 or #2 or #3 or #4 or #5 or #6 or #7 or #8 or #9 or #10 or #11 or #12 or #13 or #14 or #15 or #16 or #17 or #18 or #19	171531
#21	MeSH descriptor: [Acceptance and Commitment Therapy] explode all trees	281
#22	(acceptance* near/3 (commit* or mind* or base* or focus* or intervention* or therap* or treat*))	2898
#23	#21 or #22	2898
#24	#20 and #23	324
	with Cochrane Library publication date from Aug 2019 to Jun 2022	132

Apply limits > Select limits > Run search

4.4 ASSIA via ProQuest

MAINSUBJECT.EXACT.EXPLODE("Obesity") OR MAINSUBJECT.EXACT.EXPLODE("Body weight") OR MAINSUBJECT.EXACT.EXPLODE("Body Mass Index") OR MAINSUBJECT.EXACT.EXPLODE("Feeding patterns") OR MAINSUBJECT.EXACT.EXPLODE("Caloric intake") OR obes* OR overweight OR over-weight OR (weight NEAR/3 (body or chang* or loss* or maint* or manag* or control* or reduct*)) OR (food NEAR/3 (intake or habit*)) OR ("body mass index" or bmi) OR (body NEAR/3 mass) OR (calori* NEAR/3 (restrict* or restrain* or reduc*)) OR (feeding NEAR/3 (pattern* or behavio*)) OR (diet* NEAR/3 (restrict* or restrain* or reduc*)) OR (waist* NEAR/3 circumferenc*)

AND

acceptance* NEAR/3 (commit* or mind* or base* or focus* or intervention* or therap* or treat*)

Limit by publication date (2019-09-25 to 2022-06-21)

4.5 WEB OF SCIENCE via web of science

(TS=(obes*) OR TS=(overweight or over-weight) OR TS=(weight NEAR/3 (body or chang* or loss* or maint* or manag* or control* or reduct*)) OR TS=(food NEAR/3 (intake or habit*)) OR TS=("body mass index" or bmi) OR TS=(body NEAR/3 mass) OR TS=(calori* NEAR/3 (restrict* or restrain* or reduc*)) OR TS=(feeding NEAR/3 behavio*) OR TS=(diet* NEAR/3 (restrict* or restrain* or reduc*)) OR TS=(waist* NEAR/3 circumferenc*)) AND (TS=(acceptance* NEAR/3 (commit* or mind* or base* or focus* or intervention* or therap* or treat*)))

Refined by: PUBLICATION date (2019-09-25 to 2022-06-20)

4.6 CINAHL via EBSCOhost

((MH "Obesity+") OR (MH "Body Weight+") OR (MH "Eating Behavior+") OR (MH "Body Weight Changes+") OR (MH "Weight Loss+") OR (MH "Body Mass Index") OR (MH "Waist Circumference") OR (MH "Weight Reduction Programs") OR (TX obes*) OR (TX (overweight or over-weight)) OR (TX (weight N3 (body or chang* or loss* or maint* or manag* or control* or reduct*))) OR (TX (food N3 (intake or habit*))) OR (TX (body mass index or bmi)) OR (TX (body N3 mass)) OR (TX (calori* N3 (restrict* or restrain* or reduc*))) OR (TX (feeding N3 behavio*)) OR (TX (diet* N3 (restrict* or restrain* or reduc*))) OR (TX (waist* N3 circumferenc*)))

AND

((MH "Acceptance and Commitment Therapy") OR (TX (acceptance* N3 (commit* or mind* or base* or focus* or intervention* or therap* or treat*))))

Limiters - Published Date: 20190801-20220631

4.7 AMED via Ovid

exp Obesity/ or exp Body Weight/ or exp Body Mass Index/ or exp Weight Loss/ or obes*.mp or (overweight or over-weight).mp or (weight adj3 (body or chang* or loss* or maint* or manag* or control* or reduct*)).mp or (food adj3 (intake or habit*)).mp or (body mass index or bmi).mp or body adj3 mass.mp or (calori* adj3 (restrict* or restrain* or reduc*)).mp or feeding adj3 behavio*.mp or (diet* adj3 (restrict* or restrain* or reduc*)).mp or (waist* adj3 circumferenc*).mp

AND

(acceptance* adj3 (commit* or mind* or base* or focus* or intervention* or therap* or treat*)).mp

Limit NUM to 2018-Current

4.8 PSYCINFO via ProQuest

MAINSUBJECT.EXACT.EXPLODE("Obesity") OR MAINSUBJECT.EXACT.EXPLODE("Body Weight") OR MAINSUBJECT.EXACT.EXPLODE("Body Mass Index") OR MAINSUBJECT.EXACT.EXPLODE("Eating Behavior") OR MAINSUBJECT.EXACT.EXPLODE("Food Intake") OR

MAINSUBJECT.EXACT.EXPLODE("Diets") OR obes* OR overweight OR over-weight OR (weight NEAR/3 (body or chang* or loss* or maint* or manag* or control* or reduct*)) OR (food NEAR/3 (intake or habit*)) OR (“body mass index” or bmi) OR (body NEAR/3 mass) OR (calori* NEAR/3 (restrict* or restrain* or reduc*)) OR (feeding NEAR/3 (pattern* or behavio*)) OR (diet* NEAR/3 (restrict* or restrain* or reduc*)) OR (waist* NEAR/3 circumferenc*)

AND

MAINSUBJECT.EXACT.EXPLODE(“Acceptance and Commitment Therapy”) OR acceptance* NEAR/3 (commit* or mind* or base* or focus* or intervention* or therap* or treat*)

Filtered by 2019-09-25 - 2022-06-21

5.0 Data extraction form

Study information

ID of data extractor	
Covidence ID	
References (Author, Publication year, Titles)	
Trial ID (if provided)	

Study characteristics

Methods

Design (e.g. randomised or not, number of trial arms, pilot or not)		
Setting (e.g. clinical, commercial, workplace, etc.)		
Country		
Inclusion/exclusion criteria	Inclusion criteria	
	Exclusion criteria	
Inclusion based on specific population characteristic? (e.g. pre-existing condition, gender, ethnicity)		
Funding statement (copy verbatim)		
Declarations of interest statement (copy verbatim)		
Any other notes to be included in characteristics of included studies table		

Participants (at baseline)

Total N randomized	
N per arm/group (where relevant)	
Total % Female	
Total mean age	
Total mean baseline BMI	
Total Ethnicity	
Total Education	
Total Socioeconomic status	
Total mean baseline EBT (rename and add as appropriate)	

Interventions (where multiple arms, copy and paste for each arm)

Any shared aspects between intervention and control groups	Target behaviour (e.g. physical activity or nutrition)	
	Mode of Delivery (e.g. group vs individual, online vs in person)	
	Duration & Frequency & Intensity (Dose)	
	Shared Intervention Content/ Components	
Comparison Arm *(rename as needed)*	Control type (e.g. waitlist / inactive, minimal intervention, usual care, standard behavioural intervention)	
	Target behaviour (e.g. physical activity or nutrition)	
	Mode of Delivery (e.g. group vs individual, online vs in person)	
	Duration & Frequency & Intensity (Dose)	
	Intervention type (e.g. CBT) and components, if applicable	
	Other intervention details	
Intervention Arm *(rename as needed)*	Target behaviour (e.g. physical activity or nutrition)	

	Mode of Delivery (e.g. group vs individual, online vs in person)	
	Duration & Frequency & Intensity (Dose)	
	ACT components (add key terms – see table on page 4)	
	Any other behaviour change strategies or intervention components	
	Other intervention details	

Mediators/Outcomes

How EBTs were measured/defined (which questionnaire was used)	
When EBTs were measured	
How weight outcome was measured (e.g. self-report vs objective etc.)	
When outcome was measured	

Outcomes (Only extract if IPD is not provided)

	Effect measure (replace as appropriate)	N (describe if taken from anywhere else other than results table)
**EBT** (mean, SD or change score) at end of intervention (please list all availiable EBTs)		
**EBT** (mean, SD or change score) at follow-up		
**Weight** (please specifiy what weight outcome used) at baseline (mean, SD)		
**Weight** (please specifiy what weight outcome used) at end of intervention (mean, SD or change score)		
**Weight** (please specifiy what weight outcome used) at follow-up (mean, SD or change score)		

6.0 Reasons for non-provision of IPD

Table SM 6-1: Reasons for not providing IPD and exclusion

Study	Reason	Citation(s)
Afari et al. (2019)	Lost contact	[1–3]

7.0 Risk of Bias assessments

7.1 RoB assessment of studies providing IPD

Figure SM 7-1: Risk of Bias assessment of included studies using a modified version of the Cochrane Risk of Bias tool 2 (RoB2)

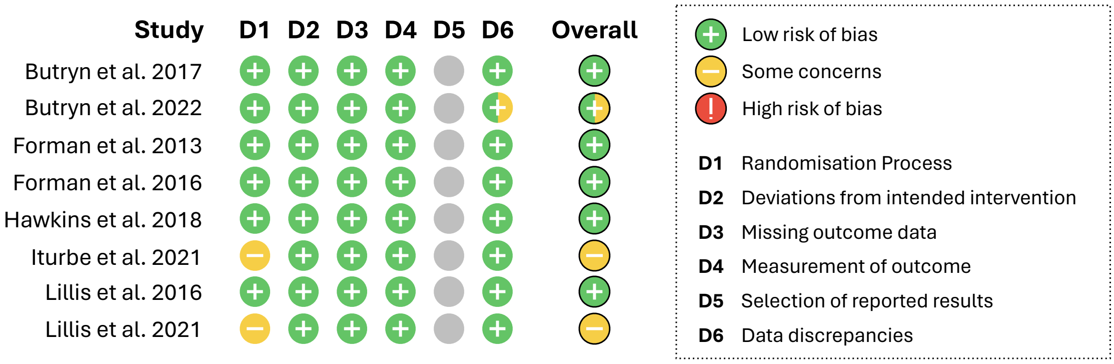

Note: Butryn 2022 was classified as some concerns only for the emotional eating outcome due to data deviations (D6)

7.2 RoB assessment of studies not providing IPD

Figure SM 7-2: Risk of Bias assessment of eligible studies that did not provide IPD using a modified version of the Cochrane Risk of Bias tool 2 (RoB2)

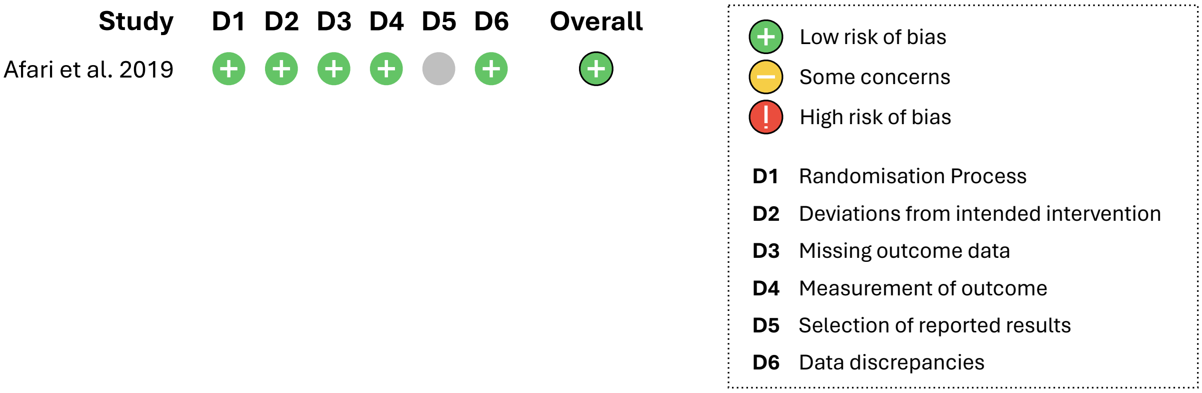

8.0 Study characteristics

8.1 Study characteristics of studies providing IPD

Table SM 8-1: Detailed characteristics of included studies that provided IPD as extracted from published manuscripts

Study	Methods	Participant characteristics	Intervention and comparators	Exposures, outcomes and time points
Butryn et al. 2017[4]	Study design:<br><br>Three-arm RCT (comparison arm merged for meta-analyses)<br><br>Country:<br><br>USA<br><br>Eligibility:<br><br>Adults (≥18y ≤70y) with a BMI between 27 and 45kg/m^2	Sex: 78.8% female<br><br>Age in years[1]:<br><br>SBT = 53.02 (9.32)<br><br>SBT + E = 53.41 (10.28)<br><br>SBT + EA = 53.23 (9.43)<br><br>BMI (kg/m^2)[1]:<br><br>SBT = 34.96 (5.19)<br><br>SBT + E = 35.38 (5.17)<br><br>SBT + EA = 35.23 (4.64)<br><br>Ethnicity:<br><br>SBT:<br><br>Black or African-American = 27%<br><br>White = 67%	Comparators:<br><br>(1) SBT (N = 88)<br><br>• **Type:** Standard behavioural treatment (SBT)<br>• **Aim:** Weight loss/ diet and physical activity<br>• **Intensity and delivery:** 26 sessions over 52 weeks with groups of 10-14 participants of 75 mins duration, delivered by clinicians with a masters or doctoral degraa in psychology and training in conducting behavioural weight loss interventions<br>• **Content:** SBT adapted from the Look AHEAD and the Diabetes Prevention Program protocols, including strategies such as self-monitoring, goal setting, problem solving,	EBT Measure(s):<br><br>• Emotional Eating (TFEQ-R18)<br>• Uncontrolled Eating (TFEQ-R18)<br>• Restraint (TFEQ-R18)<br>• Disinhibition (TFEQ-51)<br><br>Outcome Measure(s):<br><br>• Objectively measured weight<br><br>Assessment time points:<br><br>• 6 months (mid-treatment)<br>• 12 months (end of intervention)<br>• 18 months (6-month follow-up)

		Hispanic/Latino = 8%<br><br>SBT + E:<br><br>Black or African-American = 29%<br><br>White = 68%<br><br>Hispanic/ Latino = 4%<br><br>SBT + EA:<br><br>Black or African-American = 31%<br><br>White = 63%<br><br>Hispanic/Latino = 12%<br><br><br>Education:<br><br>SBT:<br><br>High school or lower = 6%<br><br>Associates degree = 16%<br><br>Bachelors degree = 36%<br><br>Graduate or professional degree = 42%<br><br><br>SBT + E:<br><br>High school or lower = 6%	identifying triggers, developing social support, relapse prevention<br><br>(2) SBT + E (N = 93)<br><br>● **Type:** SBT + content on the home environment<br>● **Aim:**<br>● **Intensity and delivery:** As above<br>● **Content:** SBT as above, with the addition of strategies including modifying the availability of foods to reduce foods promoting overconsumption and increase foods facilitating weight management, adapting home environment to increase cues of physical activity<br><br>Intervention (N = 102):<br><br>● **Aim:**<br>● **Intensity and delivery:** As above<br>● **Content:** SBT + E as above, with the addition of ACT content<br>● **ACT components:**<br>○ Acceptance<br>○ Willingness<br>○ Values Clarification<br>○ Committed Action	● 24 months (12-month follow-up)

		Associates degree = 21%<br><br>Bachelors degree = 23%<br><br>Graduate or professional degree = 50%<br><br><br>SBT + EA:<br><br>High school or lower = 7%<br><br>Associates degree = 12%<br><br>Bachelors degree = 46%<br><br>Graduate or professional degree = 35%<br><br><br>Income: not reported		
Butryn et al. (2022) [5–7]	Study design:<br><br>Three-arm RCT<br><br>Country:<br><br>USA<br><br>Eligibility:<br><br>Adults (≥18y ≤70y) with a BMI	Sex: 78.1% female<br><br>Age in years[1]: 52.72 (10.35)<br><br>BMI (kg/m^2)[1]: 35.14 (4.76)<br><br>Ethnicity:<br><br>Hispanic or Latino = 3.8%<br><br>White or Caucasian = 70.0%	In phase 1, all participants received standard behavioural treatment (SBT) adapted from the Look AHEAD15 and the Diabetes Prevention Program protocols. In phase 2 they were randomized into one of three conditions:<br><br>Comparators:<br><br>(1) SBT (N = 110)	EBT Measure(s):<br>• Emotional Eating (EOQ)<br>• Disinhibition (TFEQ-51)<br>Outcome Measure(s):<br>• Objectively measured weight

	between 27 and 45kg/m^2	Black or African American = 25.0%<br><br>More than one race = 2.8%<br><br>Asian = 1.6%<br><br>American Indian or Alaska Native = 0.6%<br><br>Education:<br><br>High school graduate or less = 5.0%<br><br>Associate's or technical degree or partial college = 17.8%<br><br>Bachelor's degree = 33.1%<br><br>Graduate degree = 44.1%<br><br>Income: not reported	• **Type:** SBT<br>• **Aim:** Weight loss/ diet and physical activity<br>• **Intensity and delivery:**<br>○ Phase 1: 16 group sessions with 12 participants over a duration of 6 months (8 weekly, 8 bi-weekly), led by counsellors with doctoral level psychology training<br>○ Phase 2: 14 continued group sessions over 12 additional months (18 in total), with 7 weekly, 4 bi-weekly, and 3 sessions in month 12, 15 and 18 plus 3 one-to-one 15-min phone calls<br>• **Content:** continued SBT, including strategies such as self-monitoring, goal setting, problem solving, identifying triggers, developing social support<br><br>(2) SBT + PA (N = 105)<br><br>• **Type:** SBT + Physical Activity Focussed<br>• **Aim:** Weight loss/ diet and physical activity<br>• **Intensity and delivery:** As above	Assessment time points:<br><br>• Baseline<br>• 6 months (mid-intervention)<br>• 12 months (mid-intervention)<br>• 18 months (end of intervention)<br>• 24 months (6-month follow-up)<br>• 36 months (18-month follow-up)

			• **Content:** focus on physical activity, incorporating techniques from the behaviour change taxonomy<br><br>Intervention (N = 105):<br>• **Aim:** Weight loss/ diet and physical activity<br>• **Intensity and delivery:** As above<br>• **Content:** SBT + Acceptance based content, with focus on physical activity<br>• **ACT components:**<br>○ Present Moment Awareness<br>○ Acceptance<br>○ Value clarification<br>○ Committed Action	
Forman et al. (2013) [8,9]	Study design:<br>Two-arm RCT<br>Country:<br>USA<br>Eligibility:<br>Adults (≥21y ≤65y) with a BMI between 27 and 40kg/m^2	Sex: 85.16% female<br>Age in years[1]: 45.69 (12.81)<br>BMI (kg/m^2)[1]: 34.10 (3.64)<br>Ethnicity:<br>Caucasian 62.3%<br>African American 24.6%<br>Asian: 1.6%	Comparator (N = 54):<br>• **Type:** Standard behavioural treatment (SBT) + cognitive behavioural model<br>• **Aim:** Weight loss/ diet and physical activity<br>• **Intensity and delivery:** 30 group sessions of 75 mins duration, over a timespan of 40 weeks (weekly during weeks 1 to 20, bi-weekly in weeks 21 to 40) led by doctoral	EBT Measure(s):<br>• Emotional Eating (DEBQ and EES)<br>• Disinhibition (TFEQ-51)<br>Outcome Measure(s):<br>• Objectively measures weight<br>Assessment time points:<br>• Baseline

		Hispanic: 3.8%<br><br>Education: not reported<br><br>Income: not reported	students or clinical psychologists with training in conducting behavioural weight loss interventions<br>● **Content:** SBT based on the LEARN and Diabetes Prevention Programm weight loss and maintenance protocols, including nutrition education, and behavioural strategies like self-monitoring, stimulus control, behavioural analysis, relapse prevention, identifying triggers, problem solving, encouraging social support + introduction of the cognitive behavioural model and cognitive restructuring<br><br>Intervention (N = 74):<br><br>● **Aim:** Weight loss/ diet and physical activity<br>● **Intensity and delivery:** As above<br>● **Content:** SBT components similar to the above (without using the cognitive behavioural model) plus ACT components adapted from treatment descriptions by Hayes<br>● **ACT components:**	● 10 weeks (early intervention)<br>● 20 weeks (mid-intervention)<br>● 40 weeks (end of intervention<br>● 66 weeks (6-months follow-up)

			○ Value identification and clarification<br>○ Present moment awareness<br>○ Observing self/ self as context<br>○ Experential and present moment awareness<br>○ Defusion (Urge surfing)<br>○ Willingness<br>○ Acceptance	
Forman et al. (2016) [10–12]	Study design:<br>Two-arm RCT<br>Country:<br>USA<br>Eligibility:<br>Adults (≥18y ≤70y) with a BMI between 27 and 50kg/m^2	Sex: 82.1% female<br>Age in years[1]: 51.64 (0.73)<br>BMI (kg/m^2)[1]: 36.93 (0.42)<br>Ethnicity:<br>Caucasian 70.5%<br>African American: 24.7%<br>Asian: 1.1%<br>Hispanic: 3.7%<br>Education: not reported<br>Income: not reported	Comparator (N = 90):<br>● **Type:** Standard behavioural treatment (SBT) + cognitive behavioural therapy (CBT)<br>● **Aim:** Weight loss/ diet and physical activity<br>● **Intensity and delivery:** 25 group sessions with 10 to 14 participants of 75 mins duration over a period of 12 months (16 weekly, 5 bi-weekly, 2 monthly, 2 bi-mothly), led by doctoral level clinicians with experience delivering behavioural weight management interventions<br>● **Content:** SBT based on the LEARN and Diabetes Prevention Programm weight loss and maintenance protocols, including nutrition and	EBT Measure(s):<br>● Emotional eating (TFEQ-18)<br>● Restraint (TFEQ-18)<br>● Uncontrolled eating (TFEQ-18)<br>● Disinhibition (TFEQ-51)<br>Outcome Measure(s):<br>● Objectively measured weight<br>Assessment time points:<br>● Baseline<br>● 6 months (mid-intervention)

			physical activity education, and behavioural strategies like SMART goals, self-monitoring, stimulus control, behavioural analysis, relapse prevention, identifying triggers, problem solving, encouraging social support + CBT including distraction and confrontation, identification of cognitive distortions, cognitive restructuring and building self-esteem<br><br>Intervention (N = 100):<br><br>● **Aim:** Weight loss/ diet and physical activity<br>● **Intensity and delivery:** As above<br>● **Content:** SBT components similar to the above (without CBT) + ACT components. Approximately 85% overlap of treatments<br>● **ACT components:**<br>○ Values clarification<br>○ Acceptance<br>○ Willingness<br>○ Present moment awareness<br>○ Cognitive defusion<br>○ Committed action	● 12 months (end of intervention)<br>● 24 months (12-month follow-up)<br>● 36 months (24-month follow-up)

Forman et al. (2021) [13]	Study design:<br><br>Ongoing 2 x 2 x 2 Factorial Trial<br><br>Country:<br><br>USA<br><br>Eligibility:<br><br>Adults (≥18y ≤70y) with a BMI between 25 and 50kg/m^2	Sex: 86.23% female<br><br>Age in years[1]: 51.84 (10.91)<br><br>BMI (kg/m^2)[1]: 35.79 (5.23)<br><br>Ethnicity: n/a<br><br>Education: n/a<br><br>Income: n/a	Comparator (N = 34):<br><br>● **Type:** Standard Behavioural Treatment (SBT) only<br>● **Aim:** Weight loss/ diet and physical activity<br>● **Intensity and delivery:** 20 remote group sessions (delivered over zoom) of 85 to 130 mins duration (depending on the number of components included) + 3 minute one-to-one consultations about weight trajectory + assignments via Google Classroom<br>● **Content:** SBT adapted from the Diabetes Prevention Programme (DPP) and Look Ahead, including nutrition and physical activity education, self-monitoring, stimulus control, goal setting, problem-solving, barriers to change, improving social support, preventing relapse<br><br>Interventions:<br><br>(1) SBT + Values (N = 32)<br><br>● **Aim:** As above<br>● **Intensity and delivery:** As above	EBT Measure(s):<br><br>● Emotional Eating (TFEQ-18 and DEBQ)<br>● Restraint (TFEQ-18)<br>● Uncontrolled eating (TFEQ-18)<br>● Disinhibition (TFEQ-51)<br><br>Outcome Measure(s):<br><br>● Self-reported weight via wireless scales<br><br>Assessment time points:<br><br>● Baseline<br>● 12 months (end of intervention<br>● 18 months (6-month follow-up)<br>● 24 months (12-month follow-up)<br>● 36 months (24-month follow-up)

			● **Content:** SBT as above plus ACT techniques as adapted from the MB-EAT program on values<br>● **ACT components:**<br>○ Value clarification<br>○ Committed Action<br><br>(2) SBT + Awareness (N = 35)<br><br>● **Aim:** As above<br>● **Intensity and delivery:** As above<br>● **Content:** SBT as above plus ACT techniques as adapted from the MB-EAT program on awareness<br>● **ACT components:**<br>○ Present moment awareness<br><br>(3) SBT + Willingness (N = 35)<br><br>● **Aim:** As above<br>● **Intensity and delivery:** As above<br>● **Content:** SBT as above plus ACT techniques as adapted from the MB-EAT program on willingness<br>● **ACT components:**<br>○ Cognitive defusion<br>○ Acceptance<br>○ Committed Action<br><br>(4) SBT + Values + Awareness (N = 38)	

			• **Aim:** As above<br>• **Intensity and delivery:** As above<br>• **Content:** SBT as above plus ACT techniques as adapted from the MB-EAT program on values and awareness<br>• **ACT components:**<br>○ Value clarification<br>○ Committed Action<br>○ Present moment awareness<br><br>(5) SBT + Values + Willingness (N = 36)<br><br>• **Aim:** As above<br>• **Intensity and delivery:** As above<br>• **Content:** SBT as above plus ACT techniques as adapted from the MB-EAT program on values and willingness<br>• **ACT components:**<br>○ Value clarification<br>○ Cognitive defusion<br>○ Acceptance<br>○ Committed Action<br><br>(6) SBT + Willingness + Awareness (N = 34)<br><br>• **Aim:** As above<br>• **Intensity and delivery:** As above<br>• **Content:** SBT as above plus ACT techniques as adapted from the MB-	

			EAT program on willingness and awareness<br>● **ACT components:**<br>○ Cognitive defusion<br>○ Acceptance<br>○ Committed Action<br>○ Present moment awareness<br><br>(7) SBT + Values + Willingness + Awareness (N = 32)<br>● **Aim:** As above<br>● **Intensity and delivery:** As above<br>● **Content:** SBT as above plus ACT techniques as adapted from the MB-EAT program on values, willingness and awareness<br>● **ACT components:**<br>○ Value clarification<br>○ Cognitive defusion<br>○ Acceptance<br>○ Committed Action<br>○ Present moment awareness	
Hawkins et al. (2018) [14]	Study design:<br>Ongoing two-arm pilot RCT<br>Country:<br>USA	Sex: 73% female<br>Age in years[1]: 45.37 (11.31)<br>BMI (kg/m^2)[1]: 35.5 (5.85)<br>Ethnicity:<br>American Indian/Alaska Native: 4.7%	Comparator (N = 54):<br>● **Type:** Standard behavioural treatment (SBT) + Cognitive behavioural therapy (CBT) elements<br>● **Aim:** Weight loss/ diet and physical activity	EBT Measure(s):<br>● Emotional Eating (EES)<br>Outcome Measure(s):<br>● Objectively measured weight

	Eligibility:<br><br>Adults (≥21y ≤65y) with a BMI between 27 and 52kg/m^2	Asian:1.9%<br><br>Black or African American: 4.7%<br><br>Multiracial: 5.6%<br><br>Other: 4.7%<br><br>Education: 76% bachelors degree or higher<br><br>Income: n/a	● **Intensity and delivery:** 23 weekly group sessions delivered over a period of 6 months<br>● **Content:** SBT based on the LEARN and Diabetes Prevention Programm weight loss and maintenance protocols, including nutrition and physical activity education, and behavioural strategies like self-monitoring, stimulus control, behavioural analysis, relapse prevention, encouraging social support + CBT including distraction and confrontation, identification of cognitive distortions, cognitive restructuring and building self-esteem (adapted from Forman et al. 2016)<br><br>Intervention (N = 54):<br><br>● **Aim:** Weight loss/ diet and physical activity<br>● **Intensity and delivery:** As above<br>● **Content:** SBT components similar to the above (without the CBT elements) + ACT<br>● **ACT components:**<br>○ Value clarification<br>○ Present moment awareness	Assessment time points:<br><br>● Baseline<br>● 6 months (end of intervention)<br>● 12 months (6-month follow-up)

			○ Cognitive defusion (urge surfing)	
Iturbe et al. (2021) [15,16]	Study design:<br>Ongoing two-arm RCT<br>Country:<br>Spain<br>Eligibility:<br>Adults (≥20y ≤70y) with a BMI ≥25kg/m^2	Sex: 70.75% female<br>Age in years[1]: 50.65 (10.66)<br>BMI (kg/m^2)[1]: 37.43 (6.77)<br>Ethnicity: n/a<br>Education: n/a<br>Income: Average annual neighbourhood income 24590.1 € (4180.8)	Comparator (N = 78):<br>● **Type:** Usual care<br>● **Aim:** Weight loss/ diet and physical activity<br>● **Intensity and delivery:** 5 monthly one-to-one sessions of 30 mins duration, delivered by a nutritionist<br>● **Content:** Nutrition counselling, including eating and physical activity-related instructions and recommendations, seasonally adapted weekly menu, nutritional labelling, recommendations for maintaining healthy habits<br><br>Intervention (N = 69):<br>● **Aim:** Health behaviours according to own values (avoiding overemphasis on weight loss)<br>● **Intensity and delivery:** 15 group sessions with 12 to 14 participants of 2hs duration (10 weekly, 5 bi-weekly), led by two psychologists with training in third-wave therapies + monthly telephone calls for a	EBT Measure(s):<br>● Emotional eating (DEBQ)<br>● Restraint (DEBQ)<br>● External eating (DEBQ)<br><br>Outcome Measure(s):<br>● Objectively measured weight<br>Assessment time points:<br>● Baseline<br>● 1 month<br>● 2 months<br>● 3 months<br>● 4 months<br>● 5 months (end of intervention)<br>● 11 months (6-month follow-up)<br>● 17 months (12-month follow-up)<br>● 29 months (24-month follow-up)

			period of 6 months after the end of active treatment<br>● **Content:** Combination of ACT- and mindfulness-based intervention following a VHL approach centering primarily on valued living<br>● **ACT components:**<br>○ Value clarification<br>○ Willingness<br>○ Cognitive Defusion (Urge surfing)<br>○ Acceptance<br>○ Observing self/ self as context<br>○ Present moment awareness<br>○ Committed Action	
Lillis et al. (2016) [17]	Study design:<br><br>Two-arm RCT<br><br>Country:<br><br>USA<br><br>Eligibility:<br><br>Adults (≥18y ≤70y) with high internal disinhibition (≥5 (women) or (≥4 (men) on the TFEQ-	Sex: 85% female<br><br>Age in years[1]: 50.2 (10.9)<br><br>BMI (kg/m^2)[1]: 37.6 (5.3)<br><br>Ethnicity:<br><br>Black = 5%<br><br>Hispanic = 6%<br><br>Asian = 1%<br><br>Caucasian = 88%	Comparator (N = 81):<br><br>● **Type:** SBT + cognitive restructuring<br>● **Aim:** Weight loss<br>● **Intensity and delivery:** approx.35 group sessions with 15 to 16 participants of 1h duration over a period of 12 months (6 months weekly, 3 months bi-weekly, 3 months monthly), run by co-leader pairs of PhD psychologists, PhD exercise physiologists and masters level nutritionists (everyone had	EBT Measure(s):<br><br>● Restraint (TFEQ-51)<br>● Disinhibition (TFEQ-51)<br>● Hunger (TFEQ-51)<br>Outcome Measure(s):<br><br>● Objectively measured weight<br>Assessment time points:<br><br>● Baseline

	51) and a BMI between 30 and 50kg/m^2	<u>Education:</u><br><br>Highschool = 7.5%<br><br>Some college/ vocational = 28%<br><br>Bachelors degree = 36%<br><br>Graduate or professional = 28%<br><br><u>Income:</u> n/a	training in ACT and experience with SBT)<br>● **Content:** SBT including self-monitoring, stimulus control, problem solving and goal setting + cognitive restructuring including stopping/ replacing negative thoughts, distraction techniques, relaxation skills, and environmental control methods<br><br><u>Intervention (N = 81):</u><br><br>● **Aim:** Weight loss<br>● **Intensity and delivery:** As above<br>● **Content:** SBT including self-monitoring, stimulus control, problem solving and goal setting + ACT<br>● **ACT components:**<br>○ Present moment awareness<br>○ Cognitive defusion<br>○ Observing self/ self as context<br>○ Acceptance<br>○ Values clarification<br>○ Committed action	● 6 months (mid-intervention)<br>● 12 months (end of intervention)<br>● 18 months (6-month follow-up)<br>● 24 months (12-month follow-up)

Lillis et al. (2021) [18]	Study design:<br><br>Ongoing two-arm RCT<br><br>Country:<br><br>USA<br><br>Eligibility:<br><br>Adults (≥25y ≤70y) with a BMI between 27.5 and 45kg/m² who lost more than 4kg in a preceding online weight loss program	Sex: 69.12% female<br><br>Age in years[1]: 57.43 (10.07)<br><br>BMI (kg/m²)[1]: 34.36 (4.32)<br><br>Ethnicity:<br><br>Black = 2%<br><br>Hispanic = 2%<br><br>Native American = 1%<br><br>Biracial = 4%<br><br>Caucasian = 91%<br><br>Education:<br><br>Highschool = 7%<br><br>Some college/ vocational = 22%<br><br>Bachelors degree = 34%<br><br>Graduate or professional = 36%<br><br>Income: n/a	In phase 1, all participants received standard behavioural treatment (SBT). In phase 2, participants who lost more than 4kg were randomized into one of two conditions:<br><br>Comparator (N = 34):<br><br>● **Type:** Extended SBT for weight loss maintenance<br>● **Aim:** Prevent weight re-gain<br>● **Intensity and delivery:**<br>○ Phase 1: 12 weekly online lessons including 10 to 15 min interactive exercises + website for self-monitoring + weekly automated feedback<br>○ Phase 2: 4 in-person group sessions with 6 to 10 participants of 2.5 hours duration over a period of 4 months after phase 1 + 6 months of weekly emails containing reminders and micro interventions (1 to 7 mins duration) + monthly feedback emails<br>● **Content:** Extends and builds on skills taught in SBT. Participants will be taught the 3 key components of self-regulation: self-observation, self-evaluation, self-reinforcement, as	EBT Measure(s):<br><br>● Restraint (TFEQ-51)<br>● Disinhibition (TFEQ-51)<br><br>Outcome Measure(s):<br><br>● Objectively measured weight<br><br>Assessment time points:<br><br>● Phase 1 Baseline<br>● Phase 2 Baseline<br>● 6 months (end of intervention)<br>● 12 months (6-months follow-up)<br>● 18 months (12-months follow-up)<br>● 24 months (18 month-follow-up)<br>● 30 months (24-month follow-up

			well as strategies to support successful implementation of these components<br><br>Intervention (N = 34):<br>● **Aim:** prevent weight re-gain<br>● **Intensity and delivery:** As above<br>● **Content:** SBT (phase1) + ACT skills for weight loss maintenance taught in the context of how they affect momentary decisions related to food and activity.<br>● **ACT components:**<br>○ Value clarification<br>○ Acceptance<br>○ Cognitive Defusion<br>○ Present moment awareness<br>○ Committed Action	

[1] mean (SD)

Abbreviations: RCT, Randomised controlled trial; BMI, Body mass index; N, Number of participants; n/a not available or not applicable; SBT, Standard Behavioural Treatment; ACT, Acceptance and Commitment Therapy; TFEQ, Three Factor Eating Questionnaire (either 18, 21, or 51 item versions respectively); EES, Emotional Eating Scale; DEBQ, Dutch Eating Behaviour Questionnaire

8.2 Study characteristics of studies not providing IPD

Table SM 8-2: Detailed characteristics of eligible studies that were excluded and did not provide IPD

Study	Methods	Participant characteristics	Intervention and comparators	Exposures, outcomes and time points
Afari et al. (2019) [1–3]	Study design:<br>Two-arm RCT<br>Country:<br>USA<br>Eligibility:<br>US veterans (≥18y ≤75y) with a BMI ≥25kg/m^2 who reported "stress-related eating" (no cut-off) and attended at least 5 of 8 sessions or the equivalent of a remote preceding usual care weight loss program (MOVE! Or TeleMOVE)	Sex: 23.9% female<br>Age in years[1]: 57.3 (9.9)<br>BMI (kg/m^2)[1]: n/a<br>Ethnicity:<br>White: 70.5%<br>African-American: 19.3%<br>Hispanic: 13.6%<br>Education:<br>Highschol: 4.5%<br>Some college: 29.5%<br>Technical/ Vocational school: 23.9%<br>Bachelors Degree: 14.8%<br>Graduate/ professional: 26.1%<br>Income: n/a	Both groups received the MOVE! treatment before being randomised to one oft wo conditions:<br>Comparator (N = 43):<br>● **Type:** Usual care + SBT<br>● **Aim:** Weight loss<br>● **Intensity and delivery:**<br>○ MOVE!: 8 weekly group sessions or 90-day TeleMOVE programme<br>○ SBT: 4 weekly group sessions of 2h duration delivered by 3 therapists (one staff psychologist, one psychology postdoctoral fellow, and one psychology masters student)<br>● **Content:** Usual care (MOVE!) containing health education, dietary and physical activity recommendations + Standard cognitive-behavioural techniques, including goal setting, self-esteem,	EBT Measure(s):<br>● Emotional eating (DEBQ)<br>● External eating (DEBQ)<br>● Restraint (DEBQ)<br>Outcome Measure(s):<br>● Either self-reported or objectively measured weight<br>Assessment time points:<br>● Baseline (after MOVE!, before ransomisation)<br>● 4 weeks (end of intervention)<br>● 3-month post-intervention follow-up<br>● 6-month post-intervention follow-up

			self-monitoring, changing distorted thinking<br><br><u>Intervention (N = 45):</u><br>● **Aim:** Weight loss + disinhibited and binge eating<br>● **Intensity and delivery:** As above but stressed importance of at-home assignments and delivered by 4 therapists, one staff psychologist, two psychology postdoctoral fellows and one psychology masters student. Therapists had all received ACT training and attended weekly supervision<br>● **Content:** Usual care (MOVE!) + ACT<br>● **ACT components:**<br>○ Present moment awareness<br>○ Willingness<br>○ Values clarification<br>○ Committed Action<br>○ Observing self/ Self as context<br>○ Cognitive defusion<br>○ Acceptance	

[1] mean (SD)

Abbreviations: RCT, Randomised controlled trial; BMI, Body mass index; N, Number of participants; n/a not available or not applicable**;** TFEQ, Three Factor Eating Questionnaire (either 18, 21, or 51 item versions respectively); EES, Emotional Eating Scale; DEBQ, Dutch Eating Behaviour Questionnaire

9.0 Number of excluded observations and reasons

Table SM 9-1: Number of excluded observations and reasons for exclusion as indicated by original study authors

Study	Group	N	Excluded reason
Butryn 2017	Control	6	No longer meeting eligibility criteria [a]
	Intervention	3	No longer meeting eligibility criteria [a]
Butryn 2022	Control	2	No longer meeting eligibility criteria [a]
	Intervention	1	No longer meeting eligibility criteria [a]
Forman 2013	Control	0	-
	Intervention	0	-
Forman 2016	Control	0	-
	Intervention	0	-
Hawkins 2018	Control	0	-
	Intervention	0	-
Iturbe 2021	Control	0	-
	Intervention	1	Biologically implausible values
		1	No longer meeting eligibility criteria [a]
Lillis 2016	Control	0	-
	Intervention	0	-
Lillis 2021	Control	0	-
	Intervention	0	-
**Total**	Control	**8**	No longer meeting eligibility criteria [a]
	Intervention	**5**	No longer meeting eligibility criteria [a]
		**1**	Biologically implausible values

Abbreviations: N, Number of observations

Note: All exclusions other than for the reason of biologically implausible values were performed by original trial authors and carried over to the IPD dataset.

[a] Participants were excluded by original trial authors if they would no longer have met respective eligibility criteria, for example due to pregnancy

10.0 Descriptive participant characteristics

Table SM 10-1*: Participant characteristics (sex, age, baseline weight) of included studies as derived from individual participant data*

Study	Group	N	Sex/ gender		Age in years		Baseline weight in kg	
			%female	N missing	Mean (SD)	N missing	Mean (SD)	N missing
Butryn 2017	Control	175	76%	0	54 (9.45)	1	96.9 (18.07)	0
	Intervention	99	82%	0	53 (9.4)	1	96.47 (15.81)	0
Butryn 2022	Control	213	78%	0	53 (10.34)	0	96.84 (16.36)	0
	Intervention	104	78%	0	53 (10.3)	0	98.61 (18.67)	0
Forman 2013	Control	54	81%	0	45 (12.77)	0	92.03 (15.08)	0
	Intervention	74	88%	0	46 (12.91)	0	94.62 (15.32)	0
Forman 2016	Control	90	82%	0	52 (10.16)	0	101.46 (19.32)	0
	Intervention	100	82%	0	52 (9.97)	0	100.01 (18.52)	0
Hawkins 2018	Control	54	72%	0	46 (11)	0	99.3 (20.96)	2
	Intervention	53	74%	0	45 (11.72)	0	101.25 (20.6)	0
Iturbe 2021	Control	78	67%	0	51 (10.96)	0	97.81 (19.95)	0
	Intervention	67	75%	0	50 (10.37)	0	106.03 (21.31)	0
Lillis 2016	Control	81	85%	0	50 (10.65)	0	102.2 (17.7)	0
	Intervention	81	85%	0	51 (11.25)	0	102.47 (17.27)	0
Lillis 2021	Control	34	68%	0	55 (11.11)	0	95.44 (14.88)	0
	Intervention	34	71%	0	60 (8.49)	0	97.04 (18.23)	0
**Total**	Control	779	77%	0	51 (10.77)	1	97.8 (17.91)	2
	Intervention	612	82%	0	51 (11.1)	1	99.48 (18.01)	0

Abbreviations: N, Number of; SD, Standard deviation

Table SM 10-2*: Participant weight change in included studies as derived from individual participant data*

Study	Group	N	Short-term percentage weight change [a]		Long-term percentage weight change [b]	
			Mean (SD)	N missing	Mean (SD)	N missing
Butryn 2017	Control	175	-8.2 (8.18)	26	-6.73 (7.6)	40
	Intervention	99	-8.46 (7.57)	16	-7.27 (7.47)	20
Butryn 2022	Control	213	-8.48 (8.7)	53		
	Intervention	104	-7.57 (8.52)	31		
Forman 2013	Control	54	-11.17 (7.1)	19		
	Intervention	74	-10.93 (7.7)	25		
Forman 2016	Control	90	-5.83 (8.27)	25	-5.83 (8.27)	25
	Intervention	100	-7.96 (9.8)	22	-7.96 (9.8)	22
Hawkins 2018	Control	54	-4.09 (6.35)	16		
	Intervention	53	-6.83 (8.07)	15		
Iturbe 2021	Control	78	-2.5 (5.1)	14	-2.48 (4.6)	27
	Intervention	67	-4.99 (7.55)	5	-4.38 (8.96)	15
Lillis 2016	Control	81	-6.31 (7.38)	17	-3.27 (6.48)	18
	Intervention	81	-6.14 (8.4)	18	-5.32 (8.01)	19
Lillis 2021	Control	34	-7.95 (5.74)	3	-5.77 (6.1)	5
	Intervention	34	-8.58 (6.97)	2	-8.6 (8.94)	8
**Total**	Control	779	-7 (8)	190	-5.21 (7.21)	115
	Intervention	612	-7.36 (8.2)	251	-6.65 (8.70)	84

Abbreviations: N, Number of; SD, Standard deviation

[a] as assessed from baseline to the follow-up closest to the end of intervention

[b] as assessed from baseline to exclusively 12 months after the end of intervention

Table SM 10-3: Participant changes in eating behaviour traits in included studies as derived from individual participant data

Study	Group	N	Change in Emotional eating [a]		Change in External eating/ disinhibition [a]		Change in Internal disinhibition [a]		Change in Restraint [a]		Change in Uncontrolled eating [a]	
			Mean (SD)	N missing	Mean (SD)	N missing	Mean (SD)	N missing	Mean (SD)	N missing	Mean (SD)	N missing
Butryn 2017	Control	175	-14 (24.03)	38	-10 (16.83)	39	-7 (14.66)	38	21 (19.39)	38	-9 (15.34)	38
	Intervention	99	-13 (22.15)	22	-9 (14.29)	23	-6 (13.15)	22	20 (19.32)	22	-8 (13.28)	22
Butryn 2022	Control	213	2 (14.11)	79	-6 (26.42)	71	-6 (27.45)	71	-	-	-	-
	Intervention	104	0 (16.28)	35	-12 (26.35)	35	-13 (21.44)	35	-	-	-	-
Forman 2013	Control	54	-12 (17.81)	14	-13 (29.11)	14	-14 (24.11)	14	30 (20.67)	14	-	-
	Intervention	74	-13 (24.22)	15	-20 (30.71)	15	-25 (25.16)	15	35 (22.64)	15	-	-
Forman 2016	Control	90	-7 (22.26)	28	-6 (16.62)	28	-4 (13.56)	28	15 (16.45)	28	-3 (16.99)	28
	Intervention	100	-13 (21.67)	19	-7 (15.66)	19	-8 (13.97)	19	21 (17.57)	19	-6 (14.92)	19
Hawkins 2018	Control	54	-6 (12.83)	17	-	-	-	-	-	-	-	-
	Intervention	53	-7 (16.72)	20	-	-	-	-	-	-	-	-
Iturbe 2021	Control	78	-10 (18.1)	15	-9 (11.37)	15	-	-	6 (17.37)	15	-	-
	Intervention	67	-21 (22.94)	5	-19 (16.16)	5	-	-	9 (19.03)	5	-	-
Lillis 2016	Control	81	-	-	-24 (27.34)	12	-26 (26.14)	12	29 (23.76)	12	-	-
	Intervention	81	-	-	-23 (28.53)	14	-28 (29.2)	14	24 (20.33)	14	-	-
Lillis 2021	Control	34	-	-	-20 (25.55)	2	-20 (18.2)	2	32 (20.93)	2	-	-
	Intervention	34	-	-	-24 (24.68)	2	-33 (30.05)	3	32 (19.92)	2	-	-
**Total**	Control	779	-7 (20.25)	191	-11 (22.92)	181	-11 (22.92)	165	21 (21.36)	109	-7 (16.11)	66
	Intervention	612	-11 (21.97)	116	-15 (23.34)	113	-17 (23.73)	108	23 (21.16)	77	-7 (14.13)	41

Abbreviations: N, Number of; SD, Standard deviation

[a] as assessed from baseline to the end of intervention

11.0 Intervention effects on changes in EBTs

Table SM 11-1: Intervention effects on changes in EBTs with short-term sample

Study [a]	N	Change in Emotional eating [b]		Change in External eating/ disinhibition [b]		Change in Internal disinhibition [b]		Change in Restraint [b]	
		SMD (95% CI)	R^2	SMD (95% CI)	R^2	SMD (95% CI)	R^2	SMD (95% CI)	R^2
Butryn 2017	274	-0.12 (-0.28, 0.1)	0.33	-0.07 (-0.25, 0.1)	0.30	-0.12 (-0.32, 0.02)	0.27	0.08 (-0.11, 0.27)	0.40
Butryn 2022	317	-0.04 (-0.21, 0.14)	0.27	-0.15 (-0.4, -0.01)	0.23	-0.09 (-0.37, -0.01)	0.24	-	-
Forman 2013	128	-0.14 (-0.3, 0.24)	0.31	-0.15 (-0.42, 0.17)	0.31	-0.35 (-0.56, 0.02)	0.27	0.2 (-0.01, 0.39)	0.61
Forman 2016	190	-0.24 (-0.5, -0.04)	0.30	-0.12 (-0.34, 0.08)	0.24	-0.28 (-0.48, -0.06)	0.25	0.32 (0.12, 0.56)	0.28
Hawkins 2018	107	0.01 (-0.3, 0.19)	0.35	-	-	-	-	-	-
Iturbe 2021	145	-0.20 (-0.39, 0.13)	0.35	-0.41 (-0.56, -0.04)	0.32	-	-	0.18 (-0.08, 0.4)	0.48
Lillis 2016	162	-	-	-0.07 (-0.31, 0.26)	0.16	-0.1 (-0.37, 0.23)	0.14	-0.12 (-0.33, 0.12)	0.45
Lillis 2021	68	-	-	-0.06 (-0.46, 0.44)	0.21	-0.08 (-0.53, 0.22)	0.49	0.15 (-0.18, 0.44)	0.59
**Overall**		-0.11 (-0.21, 0.01)	0.35	**-0.12 (-0.23, -0.01)**	0.26	**-0.16 (-0.3, -0.08)**	0.26	**0.18 (0.09, 0.28)**	0.41

Abbreviations: N, Number of; SMD, Standardised mean difference; CI, Confidence Interval

[a] study-specific sub-models are conducted in study subsets, while basing imputation on the overall sample. The overall model does thus not reflect a summary of individual effects

[b] as assessed from baseline to the end of intervention

Table SM 11-2: Intervention effects on changes in EBTs with long-term sample

Study [a]	N	Change in Emotional eating [b]		Change in External eating/ disinhibition [b]		Change in Internal disinhibition [b]		Change in Restraint [b]	
		SMD (95% CI)	R^2	SMD (95% CI)	R^2	SMD (95% CI)	R^2	SMD (95% CI)	R^2
Butryn 2017	274	-0.13 (-0.33, 0.08)	0.32	-0.06 (-0.26, 0.11)	0.31	-0.11 (-0.36, 0.01)	0.30	0.07 (-0.12, 0.26)	0.39
Forman 2016	190	-0.26 (-0.48, -0.01)	0.29	-0.1 (-0.35, 0.1)	0.26	-0.27 (-0.49, -0.06)	0.28	0.31 (0.07, 0.52)	0.28
Iturbe 2021	145	-0.20 (-0.4, 0.13)	0.34	-0.41 (-0.63, -0.12)	0.33	-	-	0.18 (-0.04, 0.44)	0.48
Lillis 2016	162	-	-	-0.06 (-0.35, 0.23)	0.16	-0.09 (-0.36, 0.23)	0.15	-0.13 (-0.39, 0.07)	0.45
Lillis 2021	68	-	-	-0.06 (-0.49, 0.42)	0.21	-0.08 (-0.45, 0.26)	0.49	0.15 (-0.19, 0.46)	0.59
**Overall**		-0.16 (-0.29, 0.003)	0.31	-0.09 (-0.23, 0.04)	0.27	-0.14 (-0.27, -0.01)	0.38	0.13 (0.0004, 0.22)	0.41

Abbreviations: N, Number of; SMD, Standardised mean difference; CI, Confidence Interval

[a] study-specific sub-models are conducted in study subsets, while basing imputation on the overall sample. The overall model does thus not reflect a summary of individual effects

[b] as assessed from baseline to the end of intervention

12.0 Path diagrams

Figure SM 12-1: Path diagram for short-term emotional eating model

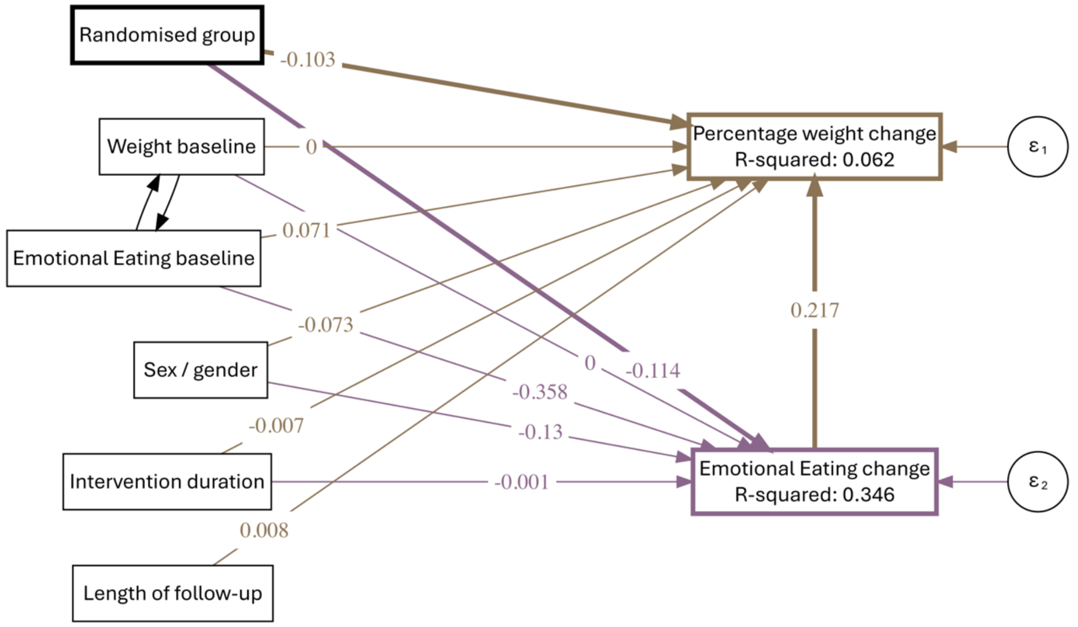

Note: Emotional eating change refers to a change from baseline to the end of intervention. Percentage weight change refers to a change from baseline to the follow-up closest to the end of intervention. Length of follow-up refers to the time from intervention end to follow-up.

Figure SM 12-2: Path diagram for long-term emotional eating model

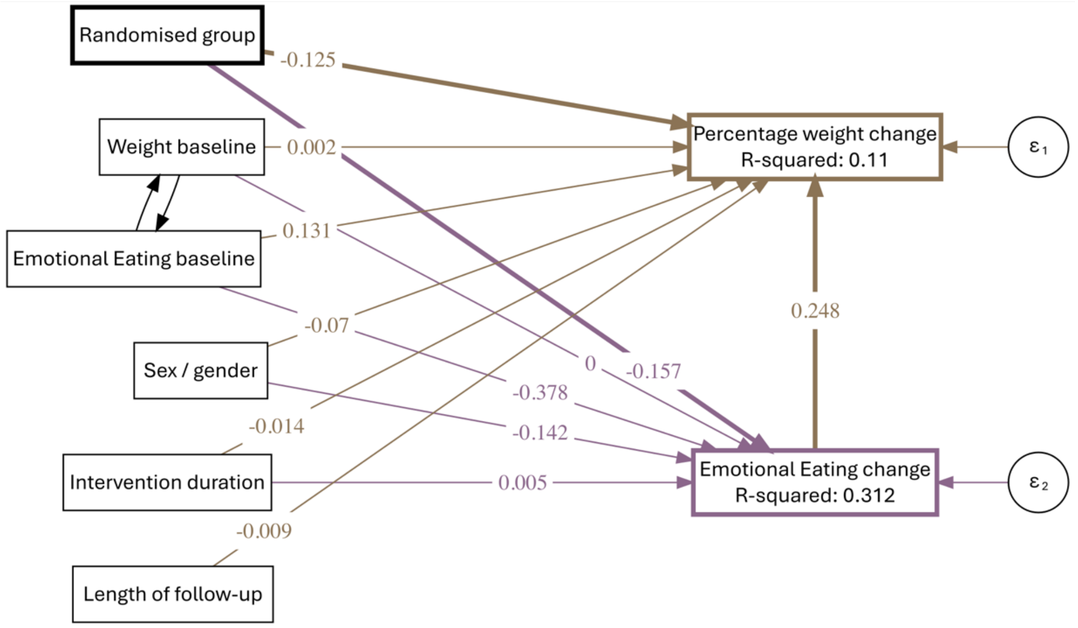

Note: Emotional eating change refers to a change from baseline to the end of intervention. Percentage weight change refers to a change from baseline to 12 months after the end of intervention. Length of follow-up refers to the time from intervention end to follow-up.

Figure SM 12-3: Path diagram for short-term external eating model

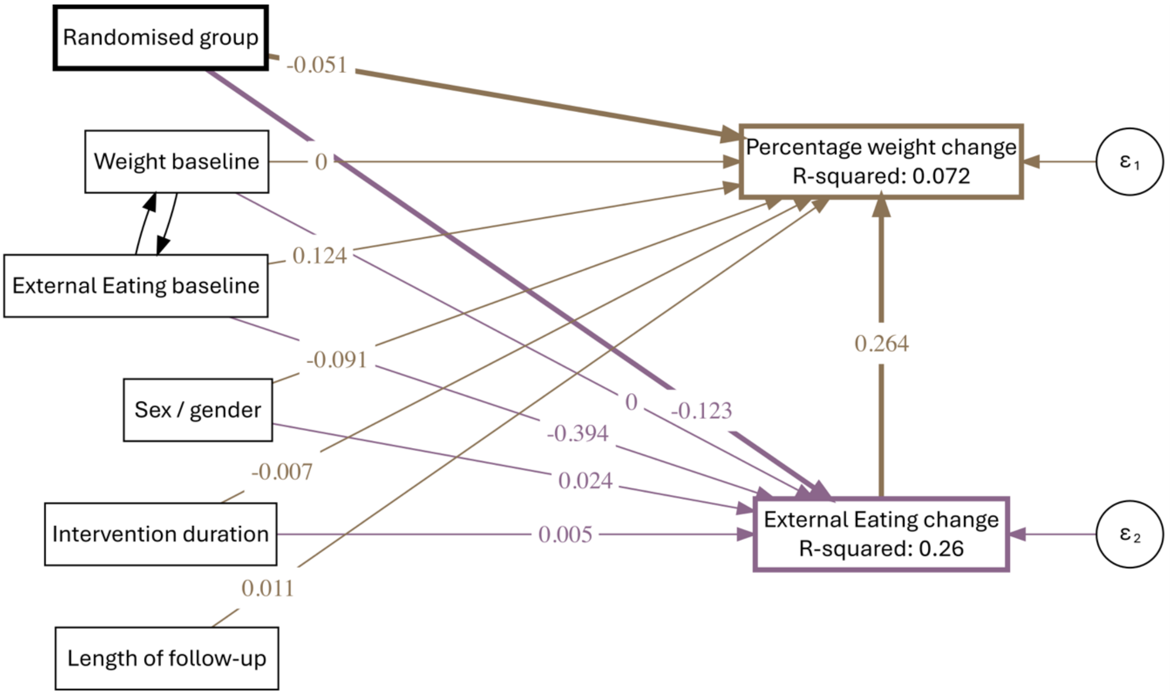

Note: External eating change refers to a change from baseline to the end of intervention. Percentage weight change refers to a change from baseline to the follow-up closest to the end of intervention. Length of follow-up refers to the time from intervention end to follow-up.

Figure SM 12-4: Path diagram for long-term external eating model

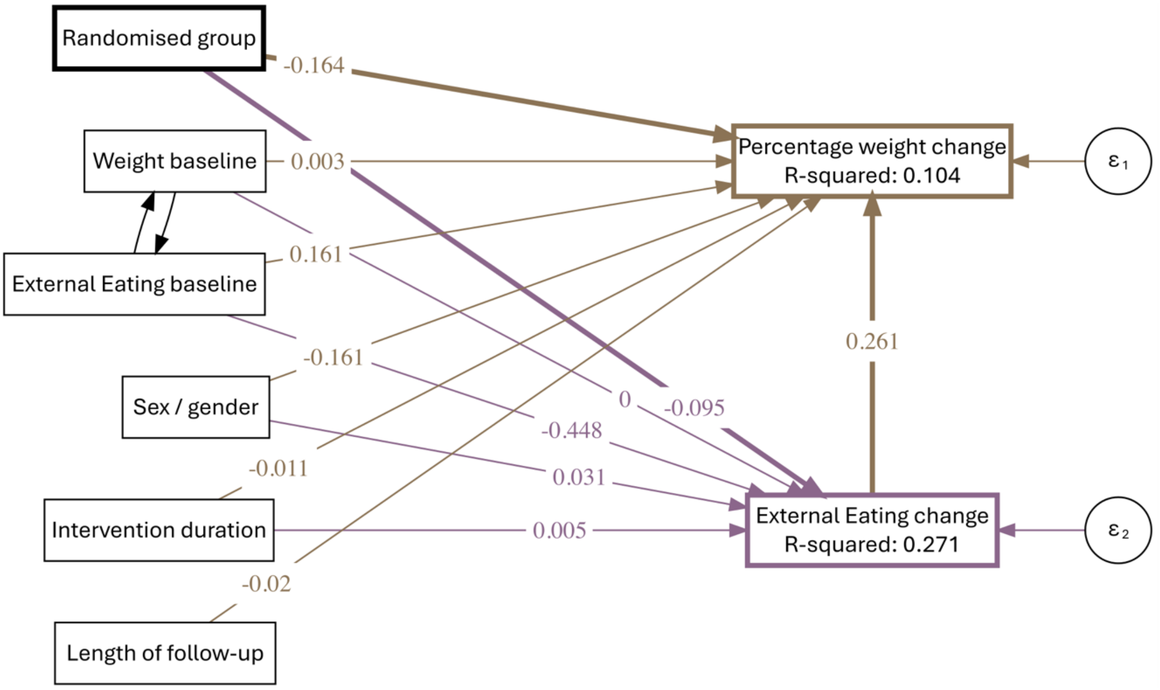

Note: External eating change refers to a change from baseline to the end of intervention. Percentage weight change refers to a change from baseline to the follow-up 12 months after the end of intervention. Length of follow-up refers to the time from intervention end to follow-up.

Figure SM 12-5: Path diagram for short-term internal disinhibition model

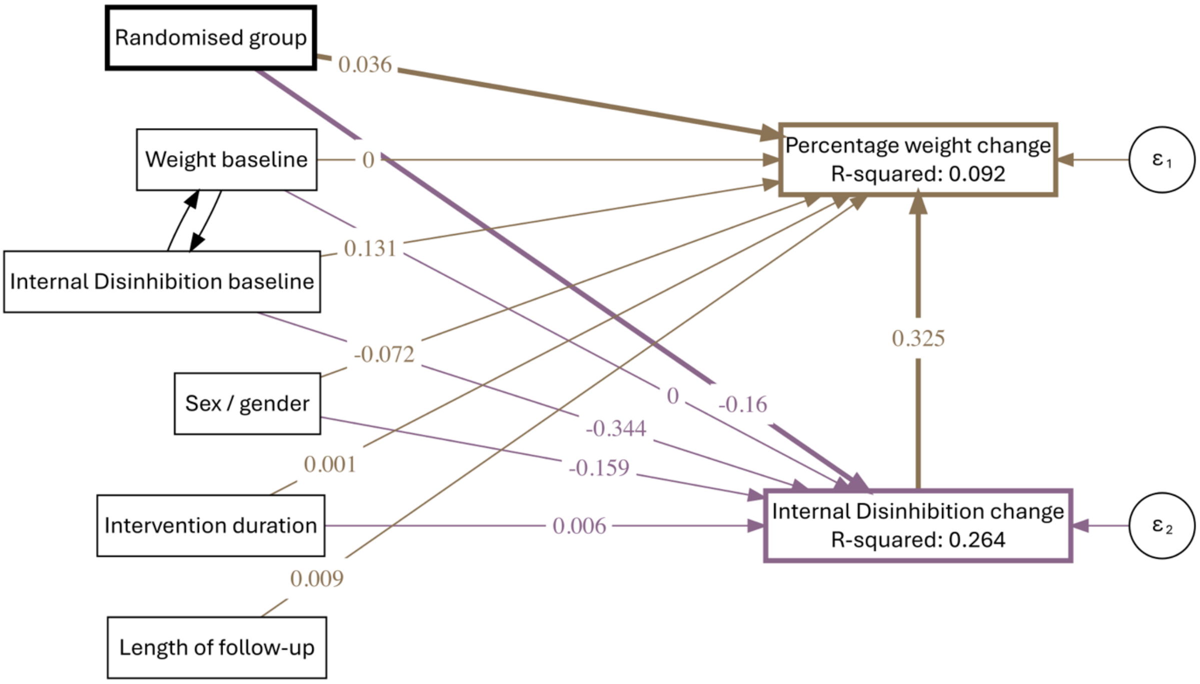

Note: Internal disinhibition change refers to a change from baseline to the end of intervention. Percentage weight change refers to a change from baseline to the follow-up closest to the end of intervention. Length of follow-up refers to the time from intervention end to follow-up.

Figure SM 12-6: Path diagram for long-term internal disinhibition model

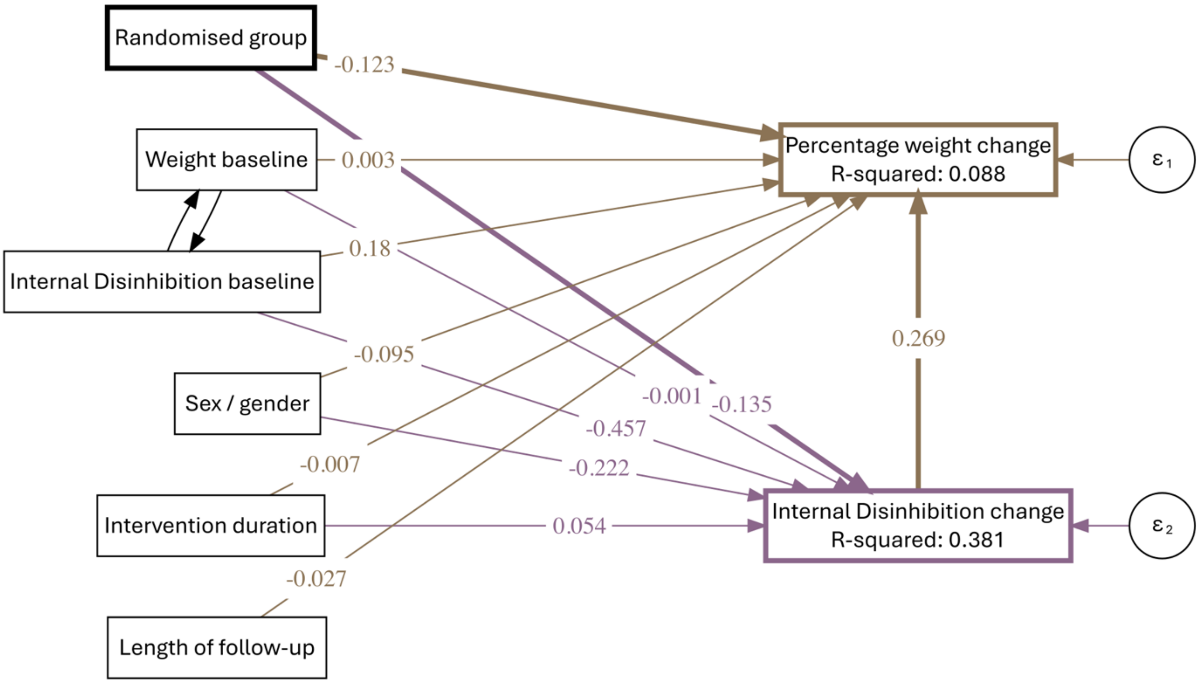

Note: Internal disinhibition change refers to a change from baseline to the end of intervention. Percentage weight change refers to a change from baseline to the follow-up 12 months after the end of intervention. Length of follow-up refers to the time from intervention end to follow-up.

Figure SM 12-7: Path diagram for short-term restraint model

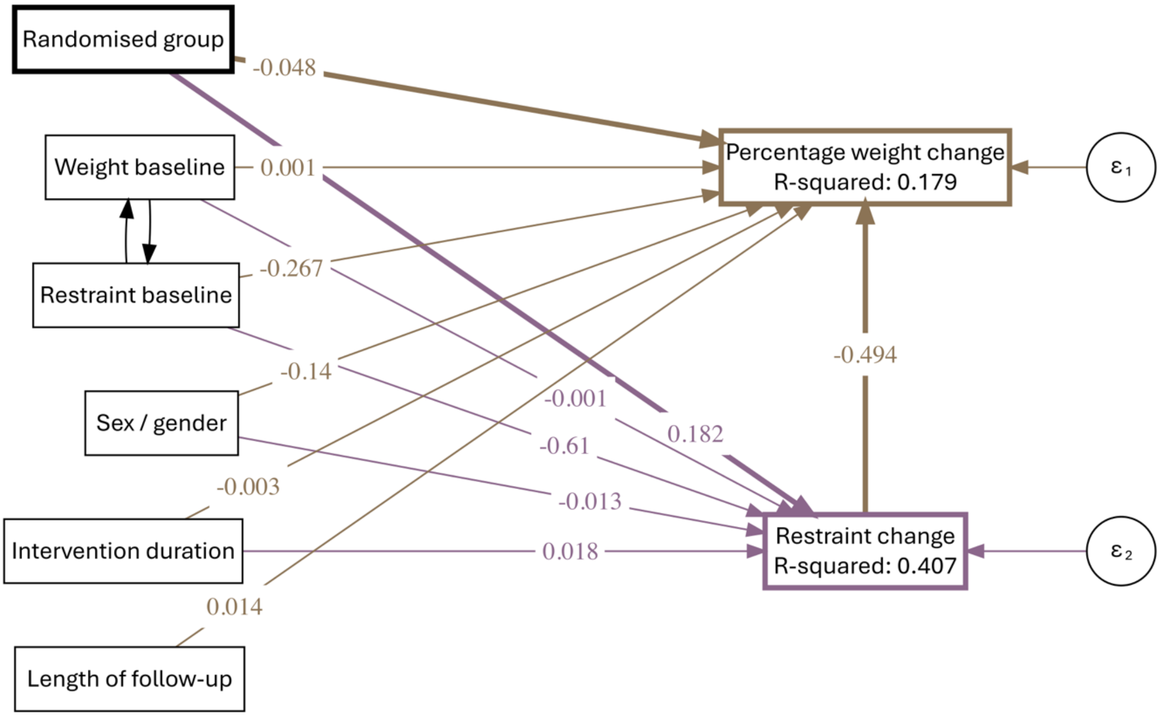

Note: Restraint change refers to a change from baseline to the end of intervention. Percentage weight change refers to a change from baseline to the follow-up closest to the end of intervention. Length of follow-up refers to the time from intervention end to follow-up.

Figure SM 12-8: Path diagram for long-term restraint model

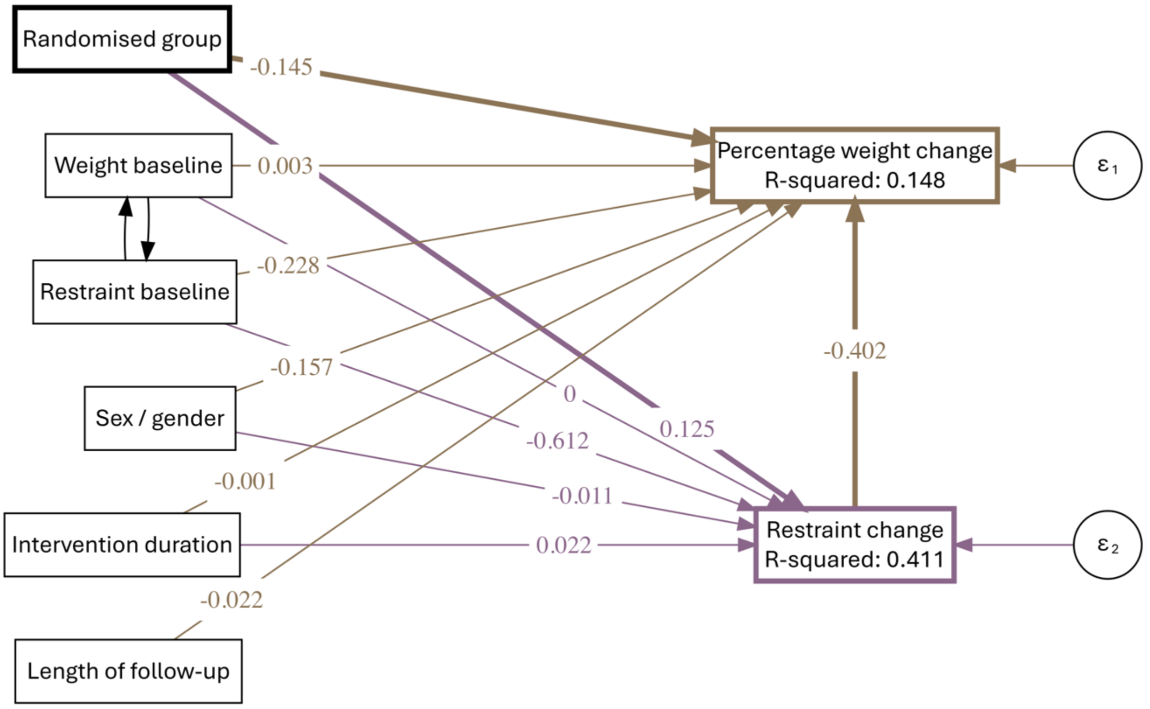

Note: Restraint change refers to a change from baseline to the end of intervention. Percentage weight change refers to a change from baseline to the follow-up 12 months after the end of intervention. Length of follow-up refers to the time from intervention end to follow-up.

13.0 Contour-enhanced funnel plots of indirect effects

Figure SM 13-1: Contour-enhanced funnel plots of indirect effects on percentage weight change through changes in eating behaviour traits in short-term mediation models

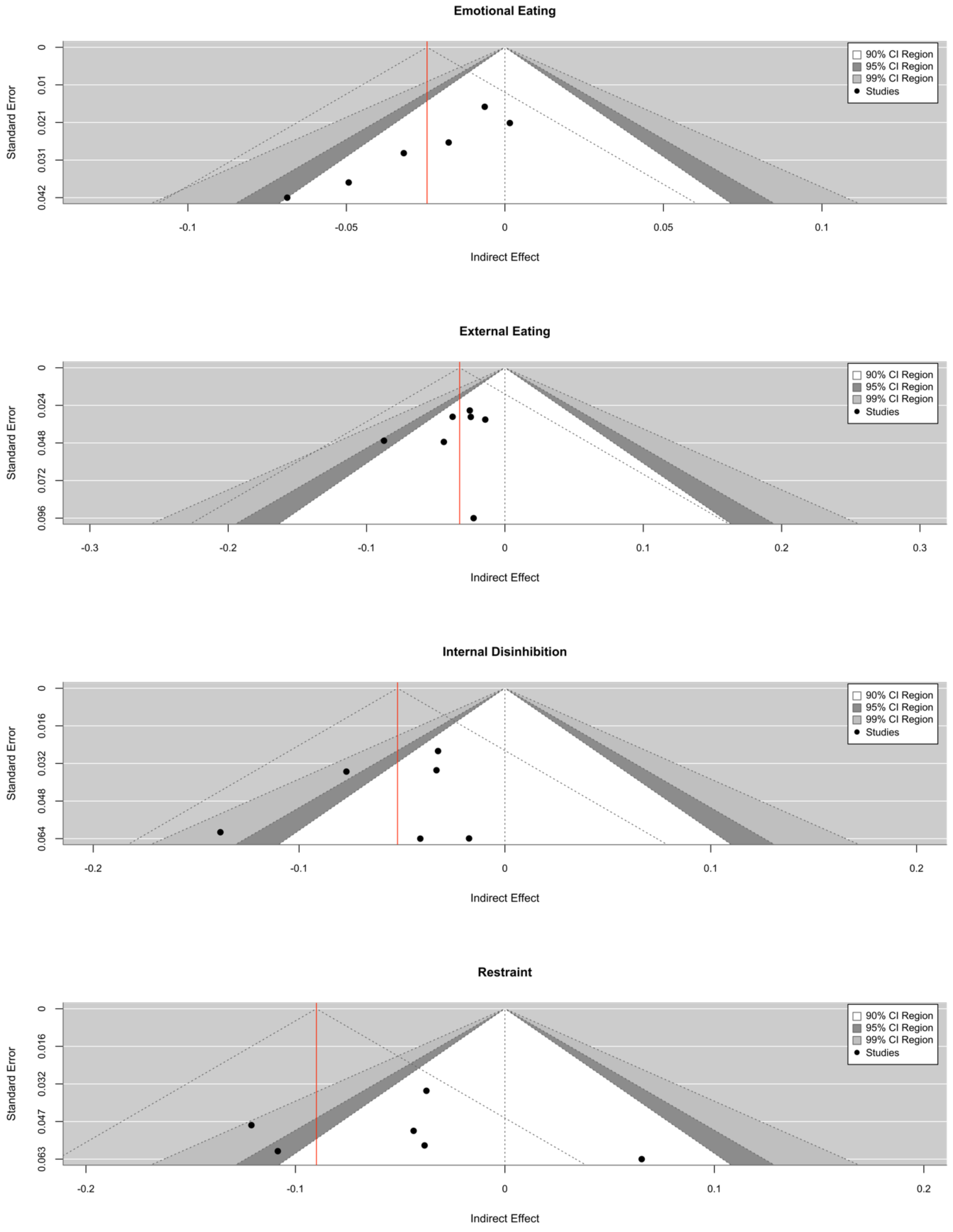

Note: The red line indicates the mean indirect effect estimate from the overall model

Figure SM 13-2: Contour-enhanced funnel plots of indirect effects on percentage weight change through changes in eating behaviour traits in long-term mediation models

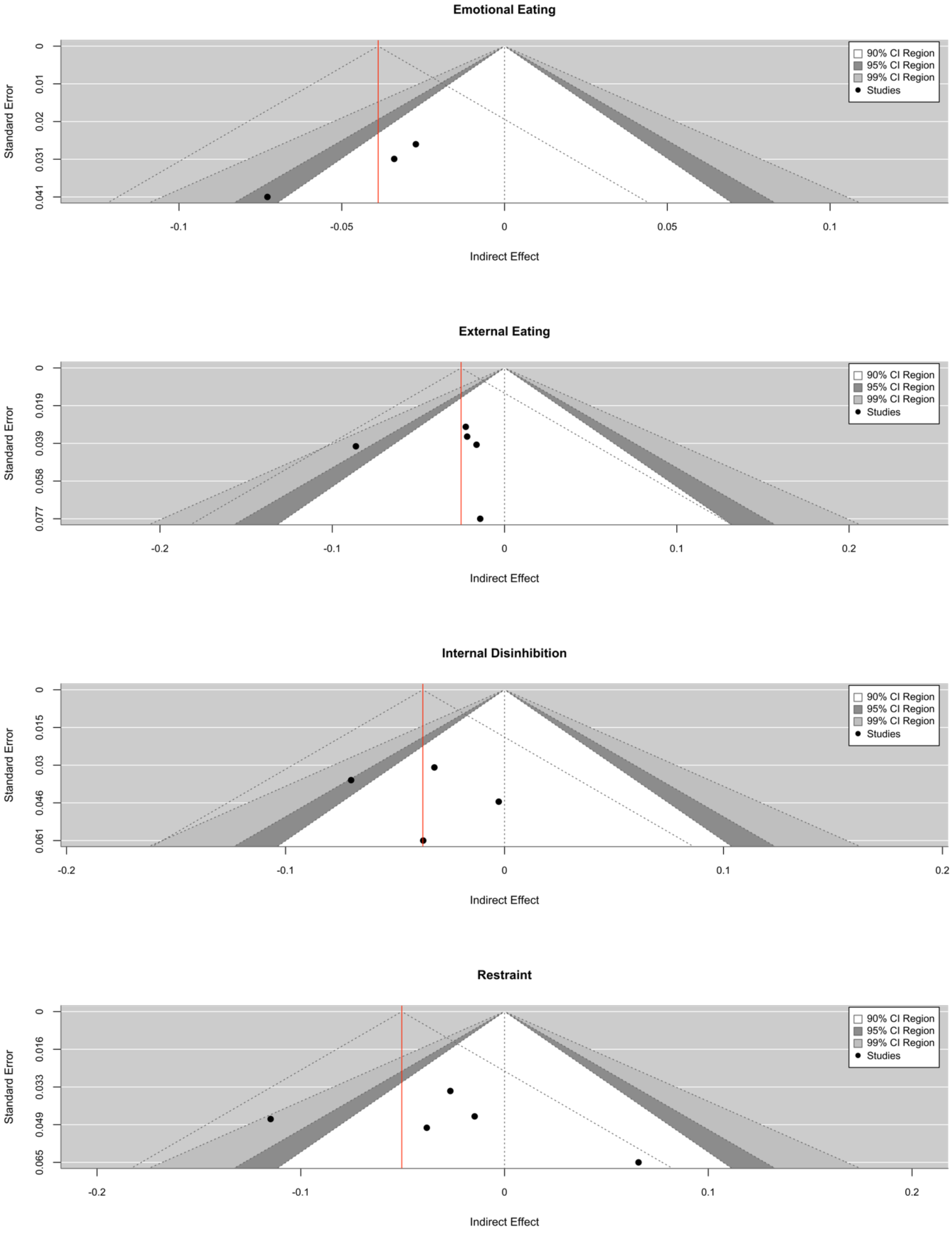

Note: The red line indicates the mean indirect effect estimate from the overall model

14.0 Sensitivity analyses of short- and long-term mediation models

Table SM 14-1: Overview of sensitivity analyses displaying the overall direct, indirect and total effects of short-term mediating models

Sensitivity analyses	N studies [N sample]	Direct effect [95% CI]	Indirect effect [95% CI]	Total effect [95% CI]
Emotional Eating				
Low risk of Bias	4 [699]	-0.133 (-0.33, 0.004)	-0.03 (-0.06, 0.02)	**-0.164 (-0.35, -0.01)**
Standard behavioural comparison	5 [1016]	-0.058 (-0.2, 0.08)	**-0.021 (-0.05, -0.001)**	-0.080 (-0.22, 0.06)
Reduced experiential avoidance	3 [592]	-0.045 (-0.24, 0.12)	-0.03 (-0.07, 0.007)	-0.08 (-0.23, 0.10)
Sufficient dose of intervention (≥ 60%)	5 [987]	-0.114 (-0.26, 0.02)	**-0.03 (-0.06, -0.004)**	**-0.143 (-0.3, -0.007)**
External Eating				
Low risk of Bias	5 [1071]	0.005 (-0.14, 0.13)	-0.024 (-0.07, 0.002)	-0.025 (-0.17, 0.11)
Standard behavioural comparison	6 [1139]	-0.003 (-0.14, 0.13)	-0.022 (-0.06, 0.001)	-0.025 (-0.17, 0.10)
Reduced experiential avoidance	3 [592]	-0.066 (-0.17, 0.17)	-0.024 (-0.06, 0.029)	-0.091 (-0.19, 0.17)
TFEQ only	6 [1139]	-0.003 (-0.14, 0.13)	-0.022 (-0.06, 0.001)	-0.025 (-0.17, 0.10)
Sufficient dose of intervention (≥ 60%)	5 [987]	-0.100 (-0.23, 0.04)	**-0.046 (-0.09, -0.01)**	**-0.147 (-0.29, -0.01)**
Internal disinhibition				
Low risk of Bias	5 [1071]	0.049 (-0.09, 0.18)	**-0.056 (-0.09, -0.01)**	-0.008 (-0.13, 0.15)
Standard behavioural comparison	6 [1139]	0.038 (-0.1, 0.16)	**-0.053 (-0.1, -0.02)**	-0.015 (-0.15, 0.12)
Reduced experiential avoidance	3 [592]	-0.006 (-0.22, 0.15)	**-0.07 (-0.11, -0.01)**	-0.07 (-0.26, 0.11)
Sufficient dose of intervention (≥ 60%)	4 [847]	-0.014 (-0.16, 0.16)	**-0.066 (-0.13, -0.04)**	-0.08 (-0.24, 0.08)
Restraint				
Low risk of Bias	4 [754]	0.009 (-0.19, 0.11)	-0.057 (-0.12, 0.01)	-0.048 (-0.26, 0.06)
Standard behavioural comparison	5 [822]	0.002 (-0.14, 0.16)	**-0.06 (-0.12, -0.01)**	-0.058 (-0.21, 0.1)
Reduced experiential avoidance	3 [592]	0.008 (-0.15, 0.19)	**-0.10 (-0.19, -0.04)**	-0.09 (-0.28, 0.08)
Sufficient dose of intervention (≥ 60%)	4 [689]	-0.109 (-0.27, 0.05)	**-0.135 (-0.19, -0.06)**	**-0.244 (-0.39, -0.06)**

Abbreviations: N, number of; CI, Confidence Interval; TFEQ, Three Factor Eating Questionnaire

Note: Sensitivity analyses comparing all trials to those with minimal comparison groups and those using the TFEQ for emotional eating and restraint could not be explored due to an insufficient number of contributing observations (< 500)

Table SM 14-2: Overview of sensitivity analyses displaying the overall direct, indirect and total effects of long-term mediating models

Sensitivity analyses		N studies [N sample]	Direct effect [95% CI]	Indirect effect [95% CI]	Total effect [95% CI]
External Eating					
	Standard behavioural comparison	4 [694]	-0.154 (-0.33, 0.01)	-0.009 (-0.05, 0.03)	-0.163 (-0.34, 0.004)
	TFEQ only	4 [694]	-0.154 (-0.33, 0.01)	-0.009 (-0.05, 0.03)	-0.163 (-0.34, 0.004)
Internal disinhibition					
	Standard behavioural comparison [a]	4 [694]	-0.123 (-0.31, 0.04)	**-0.037 (-0.09, -0.001)**	**-0.16 (-0.34, -0.003)**
Restraint					
	Standard behavioural comparison	4 [694]	-0.126 (-0.29, 0.04)	-0.041 (-0.12, 0.002)	**-0.167 (-0.35, -0.01)**

Abbreviations: N, number of; CI, Confidence Interval; TFEQ, Three Factor Eating Questionnaire

Note: Sensitivity analyses comparing all trials to those with low risk of bias for emotional eating and restraint, all trials to those with standard behavioural comparison groups for emotional eating, all trials to those with minimal comparison groups for all EBTs, all trials to those that reduced experiential avoidance for all EBTs, all participants to those that attended more than 60% of sessions for all EBTs, and those trials that used the TFEQ for emotional eating and restraint could not be explored due to an insufficient number of contributing observations (< 500). Although trials with low risk of bias had more than 500 observations for external eating (n = 626) and disinhibition (n = 626), SEM models could not produce results. The sample size was likely insufficient to accommodate imputation of an increased number of missing values at the long-term timepoint.

[a] these were the same trials included in the main analysis of long-term models.

15.0 References Supplementary Material

1 Afari N, Herbert MS, Godfrey KM, *et al.* Acceptance and commitment therapy as an adjunct to the *MOVE!* programme: a randomized controlled trial. *Obes Sci Pract*. 2019;5:397–407. doi: 10.1002/osp4.356

2 Wooldridge JS, Blanco BH, Dochat C, *et al.* Relationships Between Dietary Intake and Weight-Related Experiential Avoidance Following Behavioral Weight-Loss Treatment. *Int J Behav Med*. 2022;29:104–9. doi: 10.1007/s12529-021-09990-0

3 Wooldridge JS, Herbert MS, Hernandez J, *et al.* Improvement in 6-min Walk Test Distance Following Treatment for Behavioral Weight Loss and Disinhibited Eating: an Exploratory Secondary Analysis. *Int J Behav Med*. 2019;26:443–8. doi: https://dx.doi.org/10.1007/s12529-019-09796-1

4 Butryn ML, Forman EM, Lowe MR, *et al.* Efficacy of environmental and acceptance-based enhancements to behavioral weight loss treatment: The ENACT trial. *Obesity*. 2017;25:866–72. doi: 10.1002/oby.21813

5 Butryn ML, Crane NT, Lufburrow E, *et al.* The Role of Physical Activity in Long-term Weight Loss: 36-month Results From a Randomized Controlled Trial. *Ann Behav Med*. 2023;57:146–54. doi: 10.1093/abm/kaac028

6 Butryn ML, Godfrey KM, Call CC, *et al.* Promotion of physical activity during weight loss maintenance: A randomized controlled trial. *Health Psychology*. 2021;40:178–87. doi: 10.1037/hea0001043

7 Call CC, D'Adamo L, Crane NT, *et al.* The relation of grit to weight loss maintenance outcomes. *J Contextual Behav Sci*. 2022;24:60–4. doi: 10.1016/j.jcbs.2022.03.008

8 Forman EM, Butryn ML, Juarascio AS, *et al.* The mind your health project: A randomized controlled trial of an innovative behavioral treatment for obesity. *Obesity*. 2013;21:1119–26. doi: 10.1002/oby.20169

9 Forman EM, Manasse SM, Butryn ML, *et al.* Long-Term Follow-up of the Mind Your Health Project: Acceptance-Based versus Standard Behavioral Treatment for Obesity. *Obesity*. 2019;27:565–71. doi: 10.1002/oby.22412

10 Forman EM, Butryn ML, Manasse SM, *et al.* Acceptance-based versus standard behavioral treatment for obesity: Results from the mind your health randomized controlled trial. *Obesity*. 2016;24:2050–6. doi: 10.1002/oby.21601

11 Coffman DL, Oliva IB, Forman EM. Does acceptance-based treatment moderate the effect of stress on dietary lapses?. *Transl Behav Med*. 2021;11:2110–5. doi: https://dx.doi.org/10.1093/tbm/ibab078

12 Godfrey KM, Schumacher LM, Butryn ML, *et al.* Physical Activity Intentions and Behavior Mediate Treatment Response in an Acceptance-Based Weight Loss Intervention. *Annals of Behavioral Medicine*. 2019;53:1009–19. doi: 10.1093/abm/kaz011

13 Forman EM, Chwyl C, Berry MP, *et al.* Evaluating the efficacy of mindfulness and acceptance-based treatment components for weight loss: Protocol for a multiphase optimization strategy trial. *Contemp Clin Trials*. 2021;110. doi: 10.1016/j.cct.2021.106573

14 Hawkins MAW, Colaizzi J, Gunstad J, *et al.* Cognitive and Self-regulatory Mechanisms of Obesity Study (COSMOS): Study protocol for a randomized controlled weight loss trial examining change in biomarkers, cognition, and self-regulation across two behavioral treatments. *Contemp Clin Trials*. 2018;66:20–7. doi: 10.1016/j.cct.2017.12.010

15 Iturbe I, Pereda-Pereda E, Echeburúa E, *et al.* The effectiveness of an acceptance and commitment therapy and mindfulness group intervention for enhancing the psychological and physical well-being of adults with overweight or obesity seeking treatment: The mind&life randomized control trial study protocol. *Int J Environ Res Public Health*. 2021;18. doi: 10.3390/ijerph18094396

16 Iturbe I, Urkia-Susin I, Echeburúa E, *et al.* An acceptance and commitment therapy and mindfulness group intervention for the psychological and physical well-being of adults with body mass indexes in the overweight or obese range: The Mind&Life randomized controlled trial. *J Contextual Behav Sci*. 2024;34:100827. doi: 10.1016/j.jcbs.2024.100827

17 Lillis J, Niemeier HM, Thomas JG, *et al.* A randomized trial of an acceptance-based behavioral intervention for weight loss in people with high internal disinhibition. *Obesity*. 2016;24:2509–14. doi: 10.1002/oby.21680

18 Lillis J, Schumacher L, Thomas JG, *et al.* Study protocol for a randomized controlled trial comparing two low-intensity weight loss maintenance interventions based on acceptance and commitment therapy or self-regulation. *Contemp Clin Trials*. 2021;103. doi: 10.1016/j.cct.2021.106327
